# SOCS5-RBMX stimulates SREBP1-mediated lipogenesis to promote metastasis in steatotic HCC with HBV-related cirrhosis

**DOI:** 10.1038/s41698-024-00545-6

**Published:** 2024-03-01

**Authors:** Youpeng Wang, Ziyin Zhao, Tingting Guo, Tiansong Wu, Mao Zhang, Dingan Luo, Kunpeng Dou, Yeni Yang, Cheng Jin, Bingyuan Zhang, Bin Zhang, Bing Han

**Affiliations:** 1 Department of Hepatobiliary and Pancreatic Surgery, the Affiliated Hospital of Qingdao University, Qingdao, China; 2 Organ Transplantation Center, The Affiliated Hospital of Qingdao University, Qingdao, Shandong China; 3 Liver Cancer Institute, Zhongshan Hospital, Fudan University, Shanghai, China; 4 College of Information Science and Engineering, Ocean University of China, Qingdao, China; 5 Institute of Medical Robotics, School of Biomedical Engineering, Shanghai Jiao Tong University, Shanghai, China

**Keywords:** Cancer metabolism, Hepatocellular carcinoma, Oncogenes

## Abstract

Abnormal lipid metabolism promotes hepatocellular carcinoma (HCC) progression, which engenders therapeutic difficulties owing to unclear mechanisms of the phenomenon. We precisely described a special steatotic HCC subtype with HBV-related cirrhosis and probed its drivers. Hematoxylin-eosin (HE) staining of 245 HCC samples revealed a special HCC subtype (41 cases) characterized by HBV-related cirrhosis and intratumoral steatosis without fatty liver background, defined as steatotic HCC with HBV-related cirrhosis (SBC-HCC). SBC-HCC exhibits a larger tumor volume and worse prognosis than non-SBC-HCC. Screening for driver genes promoting fatty acid (FA) biosynthesis in the Gao’s HBV-related cirrhosis HCC cases and GSE121248’ HBV-related HCC cases revealed that high expression of SOCS5 predicts increased FA synthesis and that SOCS5 is upregulated in SBC-HCC. Through proteomics, metabolomics, and both in vivo and in vitro experiments, we demonstrated that SOCS5 induces lipid accumulation to promote HCC metastasis. Mechanistically, through co-IP and GST-pulldown experiments, we found that the SOCS5-SH2 domain, especially the amino acids Y413 and D443, act as critical binding sites for the RBMX-RRM domain. SOCS5-RBMX costimulates the promoter of SREBP1, inducing de novo lipogenesis, while mutations in the SH2 domain, Y413, and D443 reverse this effect. These findings precisely identified SBC-HCC as a special steatotic HCC subtype and highlighted a new mechanism by which SOCS5 promotes SBC-HCC metastasis.

## Introduction

Hepatocellular carcinoma (HCC) is the third leading cause of cancer-related deaths worldwide, with more than 900,000 new cases and 800,000 deaths every year, seriously endangering the lives and health of people around the world^1^. Surgical resection is the main treatment for HCC, and postoperative recurrence and metastasis are the main factors contributing to the poor prognosis. The current view is that cancer cells first undergo unique metabolic changes to adapt to their local harsh environment, as a hallmark of cancer cells, which is called “metabolic reprogramming”^2,3^. These metabolic changes stimulate downstream signals to promote tumor invasion and metastasis^4,5^. The liver is a crucial organ for lipid metabolism, and the abnormal expression of lipid metabolism regulators runs through the whole process of HCC^6,7^. However, the specific mechanisms linking abnormal lipid metabolism to HCC metastasis remain to be fully elucidated.

In recent years, lipid metabolism in tumor progression has received more attention. During the malignant progression of cancer cells, lipid metabolism often exhibits drastic alterations. FAs provide cancer cells with energy; In addition, FA composition (saturated/unsaturated FAs) and abundance are closely related to membrane fluidity and protein dynamics to regulate tolerance to reactive oxygen species^8^. Meanwhile, FAs form lipid rafts in the cell membrane, promoting the recruitment of signaling proteins and the transduction of related cascades to regulate various carcinogenic processes^6^. HCC is typically characterized by upregulation of genes related to FA synthesis, including sterol regulatory element binding transcription factor (SREBPs), major regulators of lipogenic enzymes, which have been shown to be significantly overexpressed in HCC and to promote HCC progression^9^. However, the specific mechanisms through which HCC cells adapt and utilize lipids to promote malignant progression remain incompletely understood.

Recent studies describe a new pathological subtype from a cohort of HCC patients from the United States and Japan, steatohepatitic HCC (SH-HCC), characterized by the partial presentation of HCC pathological features of “steatohepatitis“^10,11^. Patients with SH-HCC typically have dyslipidemia, HCV infection, and fatty liver background, and this tumor type is more aggressive than non-steatotic HCC. However, there is still a lack of research coverage of this HCC subtype in China. Our study identified similar cases of intratumoral steatosis in HCC patients. Approximately 95% of cases are characterized by HBV-related cirrhosis without fatty liver, so this HCC subtype was defined precisely as steatotic HCC with HBV-related cirrhosis (SBC-HCC). Searching for driver genes that cause abnormal lipid metabolism and intratumoral steatosis of HCC may be key to clarifying the occurrence mechanism and identifying targeted therapy for SBC-HCC.

The suppressor of cytokine signaling 5 (SOCS5) belongs to the suppressor of cytokine signaling protein family and can promote HCC invasion and migration^12^. The RNA binding motif protein X-linked (RBMX) is significantly overexpressed in HCC, which can promote the proliferation and multidrug resistance of HCC cells^13^. In addition, RBMX can recognize the SREBP1 promoter region of HCC cells, which can increase the activity of the SREBP1 promoter^14^. However, the specific regulatory mechanisms of SOCS5 and RBMX on HCC lipid metabolism have not been reported.

In this study, we precisely defined SBC-HCC as a special HCC subtype in the Chinese population and highlighted a specific mechanism to promote SBC-HCC metastasis. We identity that SOCS5 may cause HCC abnormal lipid metabolism and is one of driver genes of SBC-HCC. Through proteomics, metabolomics, and both in vivo and in vitro functional analyses, we studied the specific mechanisms of SOCS5- related abnormal lipid metabolism in HCC metastasis. Using GST pulldown and co-IP experiments, we elucidated the interaction between SOCS5 and RBMX and identified crucial binding sites. Specific blockers of SOCS5-RBMX may lay the foundation for targeted therapy for SBC-HCC.

## Results

### SBC-HCC is a special HCC subtype with a worse prognosis

**Fig. 1:**
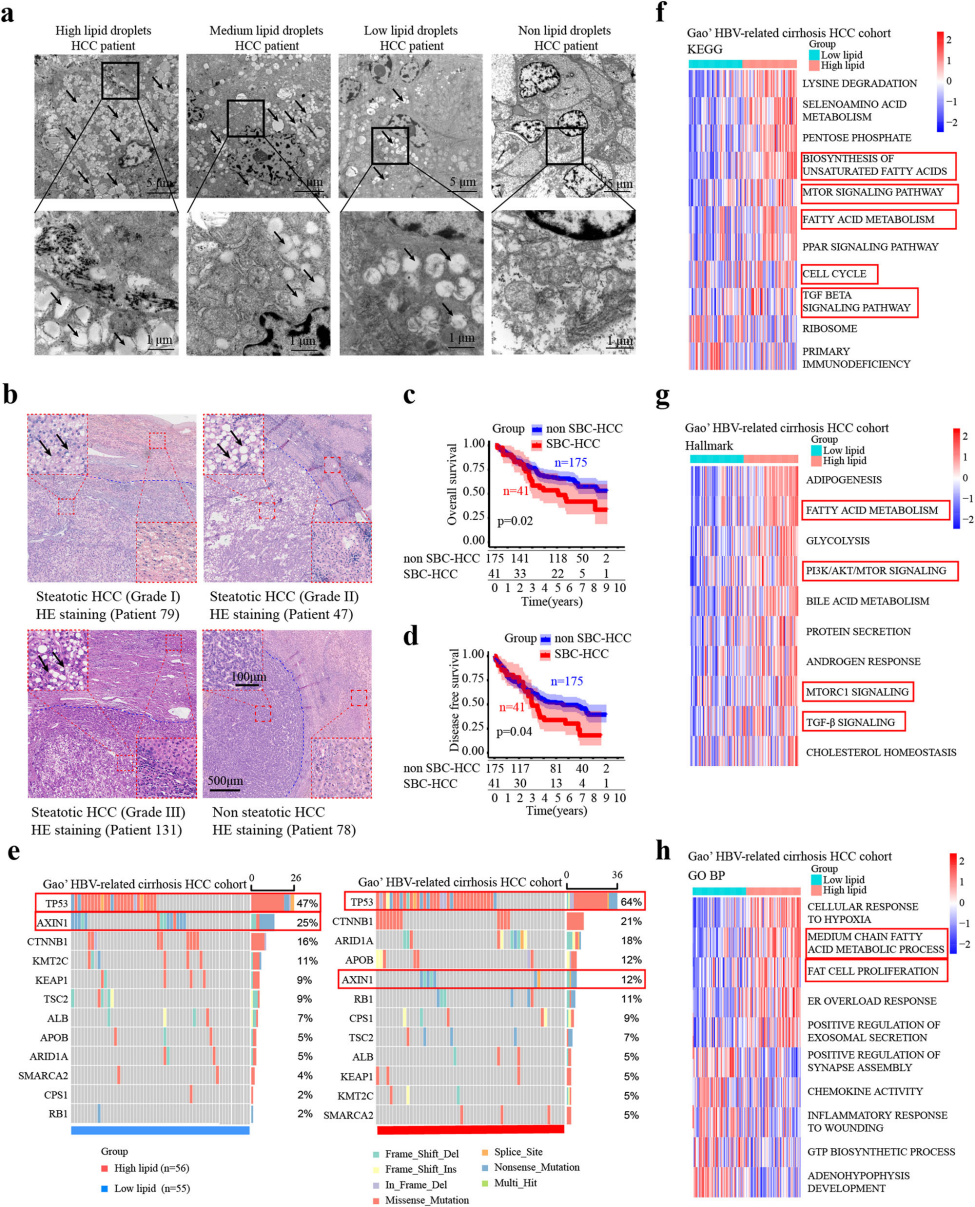
SBC-HCC predicts worse prognosis. **a** Representative electron micrographs of the LDs in HCC patients. The arrows are LDs within HCC cells. Scale bar, 5 μm and 1 μm. **b** HE staining in our cohort of 245 HCC patients; The arrows are steatotic vacuoles in the steatotic HCC group. Scale bar, 500 μm and 100 μm. **c**, **d** The prognostic value of SBC-HCC patients in our HCC cohort. *P* values were determined by the log-rank test. **e** In Gao’s HBV-related cirrhosis HCC cohort, significantly mutated genes in the high-lipid and low-lipid groups. **f**–**h** GSVA scores of patients in the high-lipid and low-lipid groups.

The volume of accumulated neutral lipids between the endoplasmic reticulum exceeds the upper limit of solubility, resulting in the formation of lipid droplets (LDs). The size and number of LDs correlate with abnormal lipid synthesis in HCC patients. By analyzing electron micrographs, we identified high-LDs, medium-LDs, low-LDs, and non-LDs HCC patients (Fig. 1a). In High-LDs HCC patients, electron micrographs reveal peripherally displaced nuclei and increased cell volume, while HE staining images display steatotic vacuoles.

To further explore the HCC subtype with abnormal lipid metabolism, we retrospectively analyzed 245 HE-stained HCC tissues in our patient cohort. Patients were divided into the steatotic HCC group (*n* = 43) and the non-steatotic HCC group (*n* = 202) based on intratumoral steatosis status (>5% tumor cells, Supplementary Table 5). 41 cases in the steatotic group met the diagnostic criteria for SBC-HCC, exhibiting intratumoral steatotic vacuoles while adjacent normal tissues did not (Fig. 1b). Moreover, 175 patients in the non-steatosis group were diagnosed with non-SBC-HCC. Compared to non-SBC-HCC cases, SBC-HCC cases presented a larger tumor volume (diameter >3 cm; Supplementary Table 1) and shorter OS and DFS (*p* < 0.05; Fig. 1c, d). Serum levels of triglyceride (TG), total bile acid (TBA), and low-density lipoprotein cholesterol (LDL-C) were significantly higher in the SBC-HCC group (*n* = 41) (*P* < 0.05; Supplementary Table 2).

### Mutational, transcriptomic, and pathological features of SBC-HCC

To explore the characteristics of SBC-HCC patients, in Gao’s HBV-related cirrhosis HCC cohort (same background as our research), patients were divided into the high-lipid (similar to SBC-HCC) and the low-lipid (similar to non-SBC-HCC) groups by their FA biosynthesis scores. GSVA analysis showed that high-lipid group had higher FA biosynthesis and cell proliferation capacity, and higher enrichment scores of TGF-β and PI3K/AKT/MTOR signaling pathways (Fig. 1f–h), accompanied by higher TP53 mutation rates (Fig. 1e). Compared with previous molecular classifications of HCC, the characteristics of SBC-HCC are similar to Zucman’s TGFβ-Wnt HCC subtype (Supplementary Fig. 1).

Interestingly, according to the diagnostic slides in the TCGA-LIHC database, we divided 369 HCC patients (follow-up time >0 days) into the steatotic HCC group (*n* = 53) and the non-steatotic HCC group (*n* = 316), and there was no difference in prognosis between the two groups (Supplementary Fig. 2a, b). Different from SBC-HCC, the TCGA HCC cohort is mainly in European and American populations with alcoholic or non-alcoholic fatty liver and HCV infection. GSVA analysis showed that the steatotic HCC patients had higher immune activation (immunological synapse formation, B cell differentiation) and lipid storage capacity, while having lower cell proliferation capacity, accompanied by lower TP53 mutation rates (Supplementary Fig. 2c–g).

The area and number of steatotic vacuoles in the SBC-HCC pathology picture (area = 10^6^ μm^2^) were further quantified, and the average steatotic vacuole area of 41 patients with SBC-HCC was 82.5 μm^2^. Area≥ 82.5 μm^2^ was defined as big steatotic vacuoles, while area <82.5 μm^2^ was defined as small steatotic vacuoles (Supplementary Fig. 3a, Supplementary Table 6). Based on the optimal cut-off value, we defined <86% (the number of small/total steatotic vacuoles) of as big steatotic vacuole SBC-HCC, and >86% as small steatotic vacuole SBC-HCC (Supplementary Fig. 3b, c). The K-M survival analysis found that small steatotic vacuoles SBC-HCC had a worse prognosis (Supplementary Fig. 3d, e).

### SOCS5 was identified as one of the driver genes for SBC-HCC

To identify the key genes involved in steatosis and lipid synthesis in SBC-HCC, we collected RNA-Seq gene expression profiles from 111 HBV-related cirrhosis HCC cases in Gao’s cohort and 107 HBV-related HCC cases from GSE121248 (Supplementary Fig. 4a). As described in the supplementary information, SOCS5 was identified as the most critical disease signature gene (Supplementary Fig. 4b–d, Supplementary Table 9). In Gao’s HBV-related cirrhosis HCC cohort, the High SOCS5 group had a higher score of FA Biosynthesis than the Low SOCS5 group (*p* < 0.05; Supplementary Fig. 4e), and the expression of SOCS5 in the High-lipid group was upregulated compared to the Low-lipid group (*p* < 0.05; Supplementary Fig. 4f).

In GSE121248, GO and KEGG enrichment analysis showed that the differentially expressed genes between the high SOCS5 group and the low SOCS5 group were closely related to FA metabolic process, FA biosynthetic process, and PPAR signaling pathway (Supplementary Fig. 5a, b). In Gao’s HCC Cohort, GSVA analysis showed that the high SOCS5 group had higher FA biosynthesis and cell proliferation capacity, and higher enrichment scores of TGF-β, PI3K/AKT/MTOR and Wnt signaling pathway (Supplementary Fig. 5c, d), accompanied by higher TP53 mutation rates (Supplementary Fig. 5e).

**Fig. 2:**
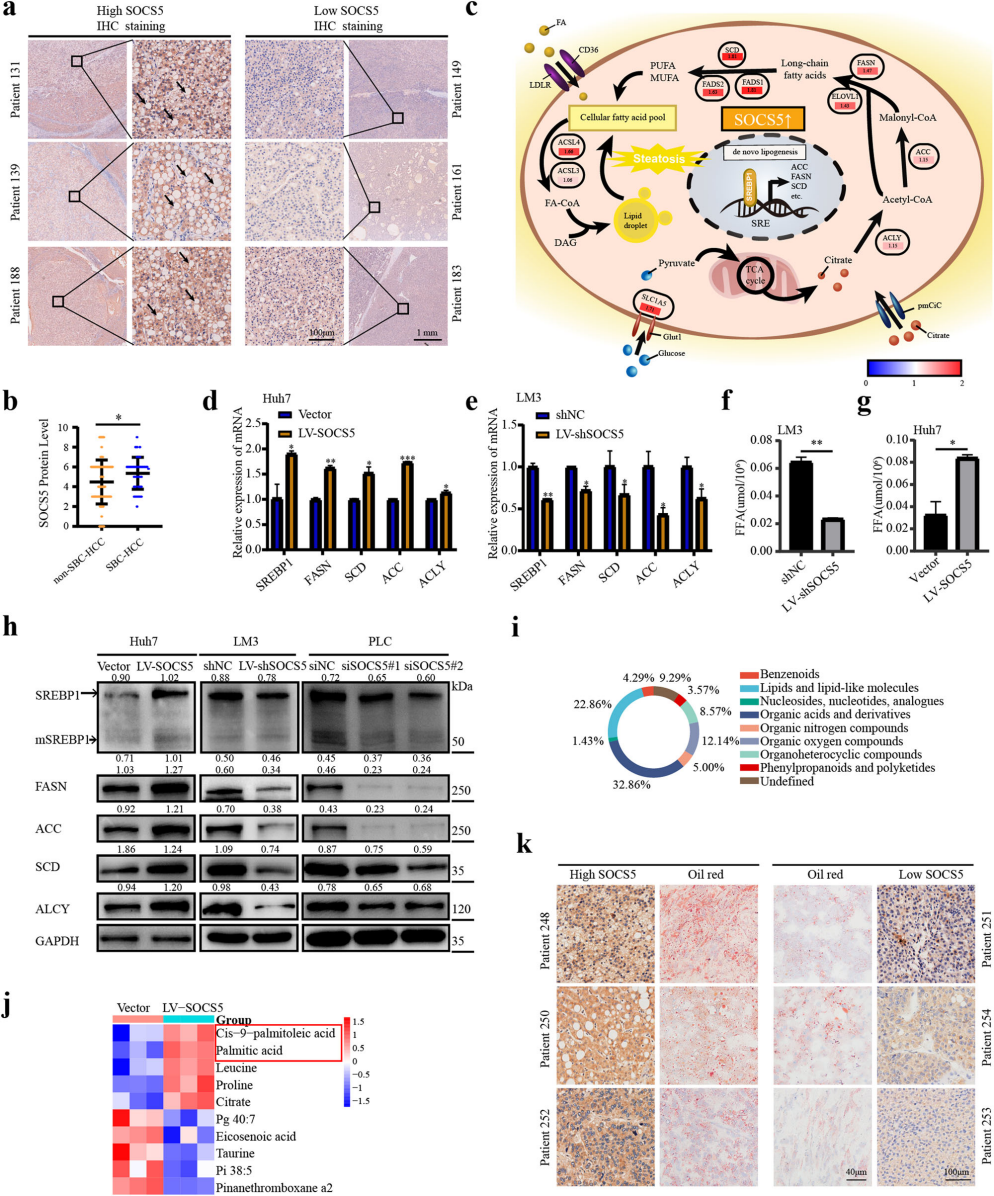
SOCS5 promotes de novo lipogenesis in HCC. **a** SOCS5 IHC staining in our cohort of 245 HCC patients; The arrows are steatotic vacuoles in the SBC-HCC group. Scale bar, 1 mm and 100 μm. **b** The expression of SOCS5 is upregulated in the SBC-HCC cohort compared to non-SBC-HCC cohort. *P* values were determined by Student’s *t* test. **c** The mechanism diagram shows that metabolism-related genes involved in the FA de novo lipogenesis are upregulated in Huh7 cells after overexpression of SOCS5. The values indicate the fold changes of metabolism-related genes after overexpression of SOCS5; Red/blue, up/down. **d**, **e** SOCS5 positively regulates the mRNA expression of metabolism-related genes involved in FA de novo lipogenesis in Huh7 and LM3 cells. *P* values were determined by the Wilcoxon test. **f**, **g** Content determination of total FFA in HCC cells after SOCS5 upregulation or downregulation. *P* values were determined by Student’s *t* test. **h** SOCS5 positively regulates the protein expression of metabolism-related genes involved in FA de novo lipogenesis in Huh7 cells. The ratios indicate SREBP1, mSREBP1, FASN, ACC, SCD, and ACLY intensity, normalized to that of GAPDH. **i** Untargeted Metabolomics of SOCS5 overexpressed Huh7, PLC, and LM3 cell lines; Chemical taxonomy information chart of all differential metabolites. **j** Heat map shows the first 5 upregulated and the first 5 downregulated metabolites in negative mode. **k** SOCS5 IHC staining in HCC tissues, and oil red staining in paired HCC tissues from the same patient. Scale bar, 100 μm and 40 μm. *P < 0.05; **P < 0.01; ***P < 0.001.

The IHC staining results of our 245 HCC patients showed that SOCS5 was expressed at a higher level in the SBC-HCC group than in the non-SBC-HCC group (*P* < 0.05; Fig. 2a, b). These data suggest that SOCS5 is closely related to abnormal lipid metabolism of HCC and may be one of the driver genes for SBC-HCC.

### SOCS5 promotes de novo lipogenesis in HCC

In our patient cohort, when compared to the SOCS5 low-expression group (*n* = 120), high expression of SOCS5 (*n* = 125) was positively correlated with steatosis, cirrhosis, and longer tumor diameters of HCC, and serum total bile acid levels were higher (Supplementary Table 3, Supplementary Table 4). Our previous research results and the Cancer Cell Line Encyclopedia (CCLE) database showed that SOCS5 had the lowest expression level in Huh7 cells compared to other HCC cells^15^ (Supplementary Fig. 6a). Therefore, in order to obtain significant and credible results, we performed proteomic analysis on HCC-Huh7 cells with SOCS5 overexpression, and 776 proteins were changed by SOCS5 overexpression (*P* < 0.05; Supplementary Fig. 6b; Supplementary Table 7). KEGG enrichment analysis showed that the differentially expressed genes were closely related to the FA biosynthesis and the PPAR signaling pathway (Supplementary Fig. 6c). The mechanistic diagram shows that genes related to de novo lipogenesis, including FASN, ACC, and SCD, are upregulated after SOCS5 overexpression (Fig. 2b). Subsequently, we overexpressed SOCS5 in Huh7 cells and knocked down SOCS5 in LM3 cells and PLC cells. We demonstrated that SOCS5 displayed a positively regulatory effect on the proteins related to de novo lipogenesis (SREBP1, mSREBP1, FASN, ACC, SCD, ACLY) in three HCC cell lines (Fig. 2d, e, h), but it did not affect plasma membrane transporters of fatty acids (CD36^16^, FATP4^17^; Supplementary Fig. 6d). Moreover, we showed that overexpression of SOCS5 can increase the content of free fatty acids (FFAs) in HCC cells, while knockdown of SOCS5 can reduce the content of FFAs (Fig. 2f, g).

Untargeted metabolomic analysis revealed that overexpression of SOCS5 in HCC Huh7 cells caused significant changes in 139 metabolites (*P* < 0.05). Among them, 22.86% of the metabolites were lipids or lipid-like molecules (Fig. 2i), including saturated FAs (palmitic acid, pentadecanoic acid, etc.) and unsaturated FAs (tridecadienoic acid, palmitoleic acid, stearidonic acid, stearic acid, etc.) (Supplementary Fig. 6f, g, Supplementary Table 8). Figure 2j and Supplementary Fig. 6e showed the top 5 upregulated metabolites and the top 5 downregulated metabolites in negative mode and positive mode according to VIP (Variable Importance for the Projection) values after SOCS5 overexpression, respectively.

In addition, through observing IHC staining and oil red O staining of HCC patients, we found that the higher the expression of SOCS5 was, the higher the content of LDs in HCC tissues (Fig. 2k).

### SOCS5 interacts with RBMX in vivo and in vitro

**Fig. 3:**
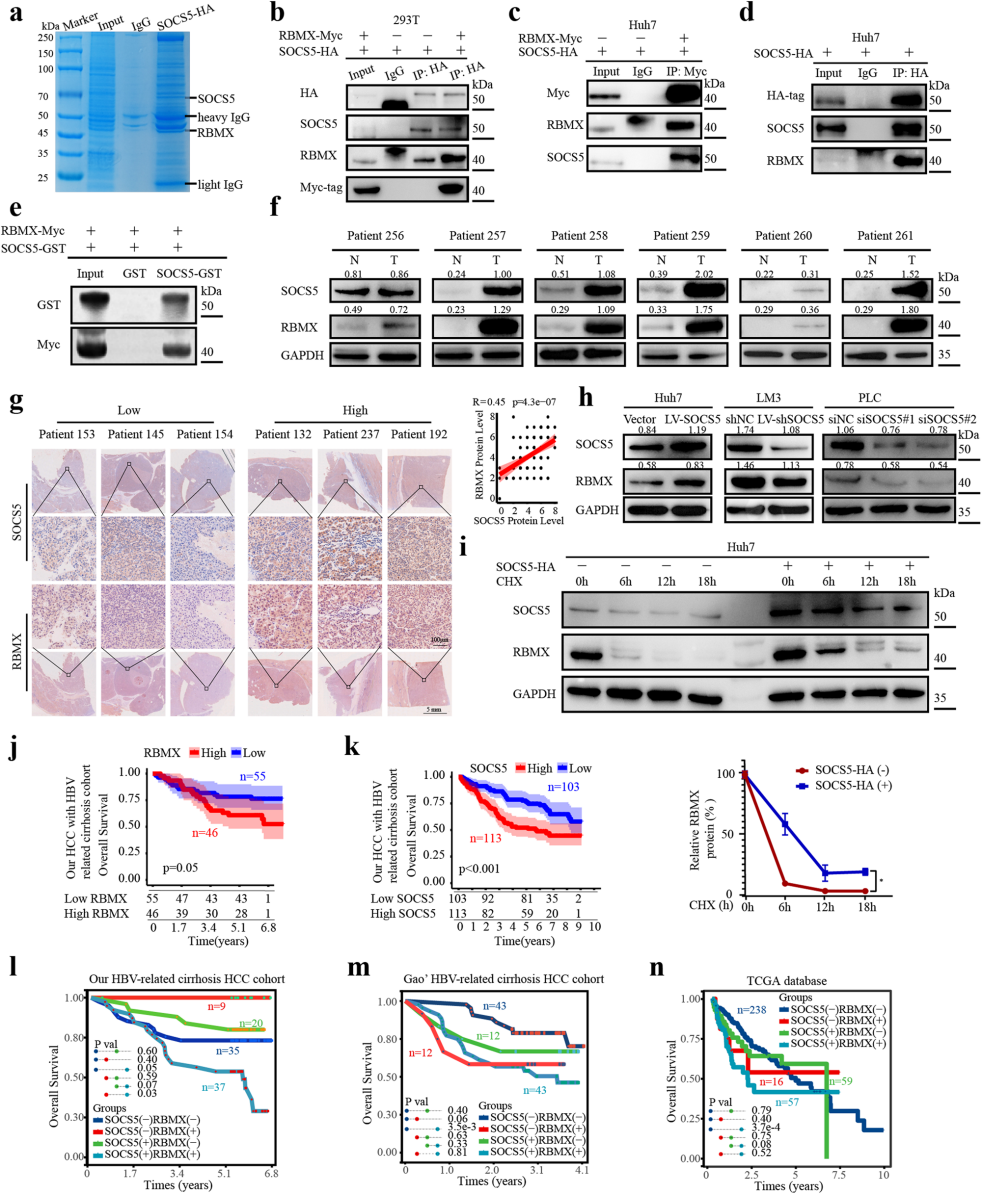
SOCS5 interacts with RBMX. **a** The immunoprecipitated proteins were visualized by Coomassie blue staining. The RBMX protein at 42 kDa may be the protein that interacts with SOCS5 at 69 kDa. **b**–**d** Co-IP experiments in 293T and Huh7 cell lines to detect SOCS5 interactions with RBMX. **e** GST pulldown assay was performed to ascertain the interaction between SOCS5 and RBMX. **f** Western blot detected protein expression levels of SOCS5 and RBMX in 6 cases of HCC tissue and paired adjacent tissues. The ratios indicate SOCS5 and RBMX intensity, normalized to that of GAPDH. **g** IHC staining of SOCS5 and RBMX and their correlation analysis in 115 HCC patients. Correlations were measured by Pearson correlation analysis. Scale bar, 5 mm and 100 μm. **h** After upregulation or downregulation of SOCS5, Western blot detects RBMX protein expression in HCC cells. The ratios indicate SOCS5 and RBMX intensity, normalized to that of GAPDH. (**i**) Representative western blots for CHX-chasing assays show that RBMX protein stability was increased in the SOCS5-HA(+) Huh7 cell line compared to the control group. **j**, **k** Kaplan–Meier overall survival curves for Low SOCS5 group (*n* = 120) and High SOCS5 group (*n* = 125), as well as the Low RBMX group (*n* = 66) and the High RBMX group (*n* = 49) in our HCC with HBV-related cirrhosis cohort. **l**–**n**) Kaplan–Meier overall survival curves of the expression combination of SOCS5 and RBMX in TCGA HCC cohort, Gao’s HBV-related cirrhosis HCC cohort, and our HBV-related cirrhosis HCC cohort. (+): high expression; (-): low expression. *P* values were determined by the log-rank test.

SOCS5 protein was labeled with HA-tag in the Huh7 cell line. SOCS5 and its binding proteins were extracted and purified by co-immunoprecipitation (co-IP) with an anti-HA antibody (Fig. 3a). Using mass spectrometry (MS), 187 proteins that might interact with SOCS5 were identified, and the top 7 potential proteins involved in lipid metabolism were selected for subsequent Western blotting verification according to the ranking of confidence (Supplementary Table 7, Supplementary Fig. 7a). Finally, RBMX was identified as the protein interacting with SOCS5.

To demonstrate the interaction of SOCS5 with RBMX, we labeled the SOCS5 protein with an HA-tag and the RBMX protein with a Myc-tag. SOCS5-HA overexpression was performed in 293T cell lines and Huh7 cell lines. Co-IP experiments were performed in Huh7 and 293T cells with anti-HA antibodies, and western blot was utilized to detected the expression of SOCS5 and RBMX. After overexpressing SOCS5-HA and RBMX-Myc in 293T cell lines, co-IP experiments were performed with anti-HA and anti-Myc antibodies, respectively. Western blot analysis showed that RBMX exhibited a higher degree of enrichment in HA-immunoprecipitated proteins after RBMX-myc overexpression compared to no RBMX-myc overexpression, and significant enrichment of RBMX, SOCS5, and Myc-tag was detected in Myc-immunoprecipitated proteins but was absent in the IgG control (Fig. 3b, c). Similarly, in Huh7 cell, significant enrichment of HA, SOCS5, and RBMX was detected in HA-immunoprecipitated proteins but not in the IgG control(Fig. 3d). A GST pulldown assay further confirmed the in vitro interaction between SOCS5-GST and RBMX-myc (Fig. 3e).

### SOCS5 increased the protein level of RBMX without affecting the RNA level of RBMX

In our patient cohort, SOCS5 and RBMX in 6 pairs of matched HCC and adjacent non-tumor frozen tissues were detected by Western blotting, and we observed that both SOCS5 and RBMX were significantly higher expressed in HCC tissues (Fig. 3f). IHC analysis of 115 HCC tissues from 2015 to 2016 showed that SOCS5 and RBMX protein expression levels were positively correlated (R = 0.45, *P* = 4.3e−07; Fig. 3g). IHC analysis of HCC tissues in the public database also showed that SOCS5 expression was positively correlated with the RBMX protein expression (Supplementary Fig. 7d).

We tested whether SOCS5 could act as a regulator of RBMX via directly binding, and found that SOCS5 can positively regulate the protein expression level of RBMX, but it seems to have no regulatory effect on mRNA level (Fig. 3h; Supplementary Fig. 7b, c). We hypothesize that SOCS5 regulates RBMX protein levels by affecting its protein stability. Therefore, we treated Huh7 cells with cycloheximide (CHX; protein synthesis inhibitor), and found that overexpression SOCS5 in Huh7 cells consistently increased RBMX stability (Fig. 3i).

According to the IHC score, 216 HCC with HBV-related cirrhosis patients from 2013 to 2016 were divided into a SOCS5 high expression group (*n* = 113) and a SOCS5 low expression group (*n* = 103). 101 HCC with HBV-related cirrhosis patients from 2015 to 2016 were divided into an RBMX high expression group (*n* = 46) and an RBMX low expression group (*n* = 55). The RBMX high expression group had a shorter OS (Fig. 3j). Similarly, 216 HCC with HBV-related cirrhosis patients from 2013 to 2016 were divided into an SOCS5 high expression group (*n* = 113) and an SOCS5 low expression group (*n* = 103). Survival analysis showed that the SOCS5 high expression group had shorter OS (Fig. 3k). Then, we analyzed the combined practical value of SOCS5 and RBMX for prognosis prediction. In our HBV-related cirrhosis HCC cohort, Gao’s HBV-related cirrhosis HCC cohort, and TCGA HCC cohort, patients in the SOCS5( + )RBMX( + ) HCC group consistently showed the shortest OS relative to SOCS5( + )RBMX(-) group, SOCS5(-)RBMX(+) group, and SOCS5(-)RBMX(-) group, especially relative to the SOCS5(-)RBMX(-) group (*P* <0.05; Fig. 3l–n).

### SOCS5 and RBMX costimulate SREBP1 transcription

**Fig. 4:**
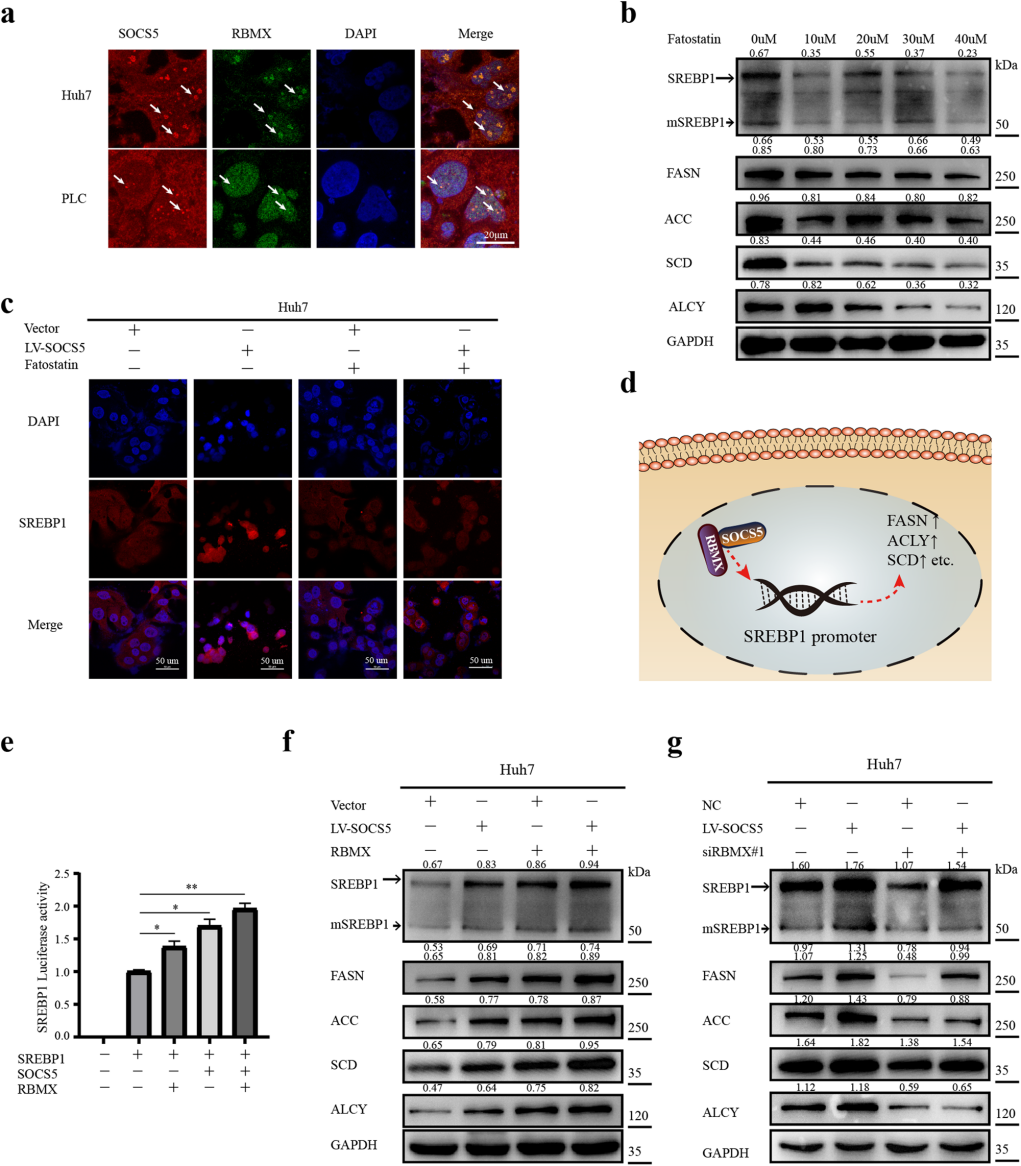
SOCS5 and RBMX costimulate SREBP1 transcription. **a** Immunofluorescent (IF) assays show colocalization of SOCS5 (red) and RBMX (green) in Huh7 cell lines and PLC cell lines. Scale bar, 20 μm. **b** Western blot analysis of SREBP1, mSREBP1, FASN, ACC, SCD, and ACLY in Huh7 cells treated with Fatostatin (0–40 μM). **c** IF analysis of protein expression and intracellular localization of SREBP1 in Huh7 cells treated with LV-SOCS5 transfection and/or Fatostatin (20 μM). Scale bar, 50 μm. **d** Schematic of SOCS5 and RBMX costimulate SREBP1 transcription. **e** The promoter luciferase activity of SREBP1 was detected by dual-luciferase reporter assay. *P* values were determined by Student’s *t* test. **f**, **g** Western blot analysis of SREBP1, mSREBP1, FASN, ACC, SCD and ACLY in Huh7 cells treated with LV-SOCS5 transfection and RBMX or siRBMX#1 transfection. The ratios indicate SREBP1, mSREBP1, FASN, ACC, SCD, and ACLY intensity, normalized to that of GAPDH. *P < 0.05; **P < 0.01; ***P < 0.001.

RBMX has been reported as a hepatic transcriptional regulator of SREBP1 gene. We further explored the regulatory effect of SOCS5-RBMX on SREBP1 and its downstream target genes. The immunofluorescence (IF) assay indicated intranuclear colocalization of SOCS5 and RBMX in Huh7 and PLC cells (Fig. 4a). As a specific inhibitor of SREBP1, Fatostatin was found to significantly inhibit the expression of SREBP1 and mSREBP1 at 20 μM, thereby significantly reducing the expression of its downstream target genes (FASN, ACC, SCD, ACLY) (Fig. 4b). Then, the IF results also showed that overexpression of SOCS5 promoted SREBP1 expression and nuclear translocation, while Fatostatin reversed this effect (Fig. 4c).

The promoter luciferase activity of SREBP1 was detected by dual-luciferase reporter assay and normalized to Renilla luciferase in 293T cell. We found that the overexpression of SOCS5 or RBMX could activate the promoter of SREBP1. Overexpression of SOCS5 and RBMX further increased SREBP1 promoter activity (Fig. 4d, e). Then, western blot experiments similarly demonstrated that overexpression of SOCS5 and RBMX further increased the expression of SREBP1, mSREBP1 and its downstream target genes (Fig. 4f), and that knockdown of RBMX (Supplementary Fig. 7e) could reverse this effect in Huh7 cell (Fig. 4g; Supplementary Fig. 7f). These results show that SOCS5, in addition to promoting SREBP1 nuclear translocation, forms a complex with RBMX in the nucleus, further activating the transcriptional effect of SREBP1 in the nucleus.

### Identify critical domains and binding sites of SOCS5-RBMX

**Fig. 5:**
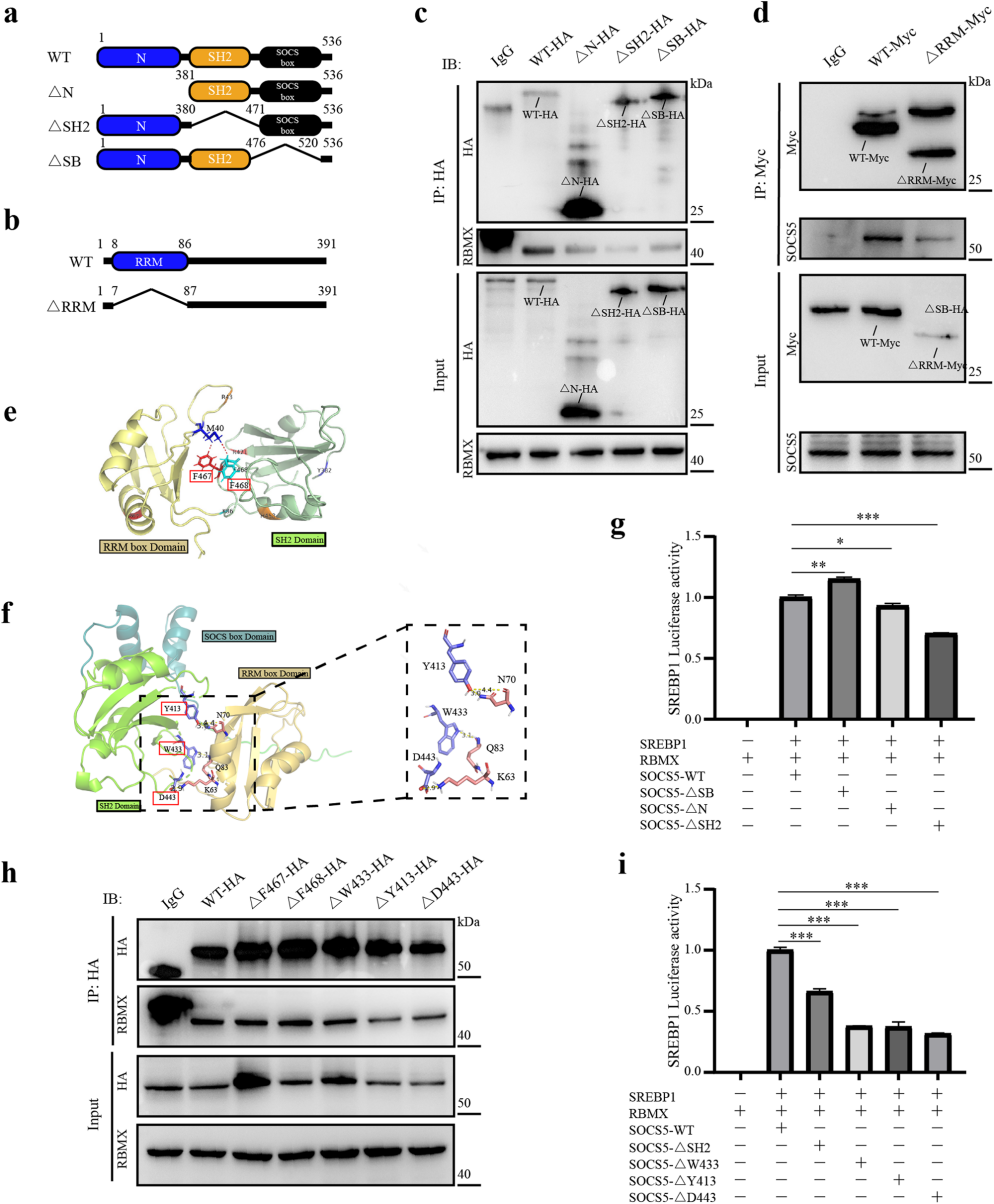
Key domains and binding sites of SOCS5-RBMX complexes. **a**, **b** Schematic of the structure of various SOCS5 mutants and RBMX mutants. **c**, **d** co-IP experiments were performed to explore the interaction between RBMX with different domain deletion mutations (WT, △RRM) and SOCS5 with different domain deletion mutations (WT, △N, △SH2, △SB). **e**, **f** Docking model analysis of the interaction between SOCS5 and RBMX by forming non-covalent bonds. The Angular step size is set to 6° and 15°, respectively. **g**, **i** The promoter luciferase activity of SREBP1 was detected by dual-luciferase reporter assay. *P* values were determined by Student’s *t* test. **h** co-IP experiments were performed to explore the interaction between SOCS5 with different point mutations (△F467, △F468, △W433, △Y413, △D443) and RBMX. *P < 0.05; **P < 0.01; ***P < 0.001.

SOCS5 is mainly composed of three domains: the N-terminal (1–380 aa), SH2 domain (381–476 aa), and SOCS Box (SB) domain (471–520 aa; Fig. 5a; Supplementary Fig. 8a). RBMX is mainly composed of RRM domains (8–86 aa; Fig. 5b; Supplementary Fig. 8b). Subsequently, we designed mutant SOCS5 and mutant RBMX domains. Co-IP experiments showed that the N-terminal domain deletion (△N) mutant and SB domain deletion mutant (△SB) slightly reduced the interaction of SOCS5 with RBMX, while the SH2 domain deletion mutation (△SH2) significantly reduced the interaction between SOCS5 and RBMX (Fig. 5c). And RRM domain deletion mutation (△RRM) significantly reduced RBMX interaction with SOCS5 (Fig. 5d). These results determined the direct binding between the SOCS5-SH2 domain and the RBMX-RRM domain.

We used two methods to study the nature of the interactions between SOCS-SH2 and RBMX-RRM. Firstly, the self-consistent calculations are preformed by DFTB embedded in QuantumATK. Three initial configurations (complex I, II and III) were prepared by molecular docking (Angular step size = 15°), and the determined E_b_ are 1.77 eV, 0.94 eV, and 1.18 eV for complex I, II and III, respectively (Supplementary Fig. 8c). According to electron density difference of complex I, the great possibility of charge transfer may take place between F467, F468 of SOCS and M40, K86 of RBMX (Fig. 5e; Supplementary Fig. 8d, e). In addition, we investigated the binding process of SOCS5-RBMX (Angular step size = 6°) and performed molecular dynamics simulations of the complexes (Fig. 5f). The Root Mean Square Fluctuation (RMSF) value showed that there were large fluctuations around ALA535, GLU529, LYS534, and LYS536 in the complex, and most amino acids fluctuated lessly (Supplementary Fig. 8h). The Root Mean Square Deviation (RMSD) mainly fluctuated between 1.8772–2.43409, and its average value was 2.25952, which was in a reasonable range, indicating that the complex was in an equilibrium after simulation (Supplementary Fig. 8f, g). LigPlot draws the eyelash map of SOCS5-RBMX interaction, and alanine virtual point mutants identified Y413, W433, D443 of SOCS5 as critical binding sites (Fig. 5f; Supplementary Fig. 8i).

Subsequently, we designed point mutants based on the predicted 5 critical binding sites of SOCS5-SH2 (F467, F468, Y413, W433, D443). Co-IP experiments showed that the W433 mutant slightly reduced SOCS5 interaction with RBMX, while the Y413 and D443 mutants significantly reduced SOCS5 interaction with RBMX (Fig. 5h). Dual-luciferase reporter assay showed that SOCS5 mutations in SH2 domain, Y413, W433 and D443 reversed the activation effect of SOCS5-RBMX on the promoter activity of SREBP1 (Fig. 5g, i).

### SOCS5 promotes SREBP1-mediated de novo lipogenesis and contributes to metastasis in HCC

**Fig. 6:**
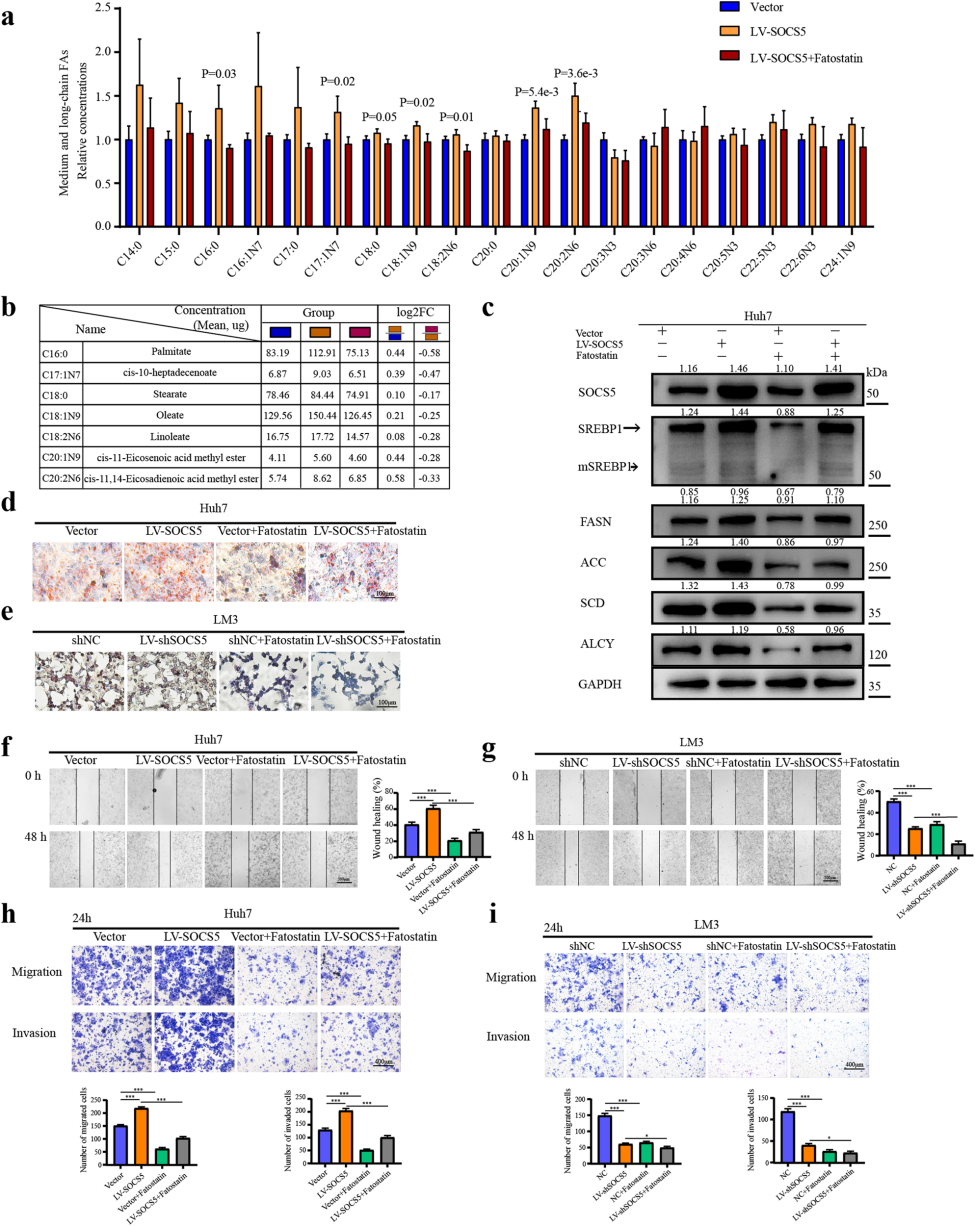
SOCS5 promotes SREBP1-mediated de novo lipogenesis and contributes to metastasis in HCC. **a** Metabolomics assays for targeted medium- and long-chain fatty acids in Huh7 cells. The results showed as vector-normalized and presented as mean ± SD. *P* values were determined by ANOVA. **b** Seven fatty acids with significant differences in concentration between the three groups (μg/10^7^ cells). **c** Western blot analysis of SREBP1, mSREBP1, FASN, ACC, SCD and ACLY in Huh7 cells treated with LV-SOCS5 transfection and/or Fatostatin (20 μM). The ratios indicate SOCS5 SREBP1, mSREBP1, FASN, ACC, SCD, and ACLY intensity, normalized to that of GAPDH. **d**, **e** Comparison of the content of intracellular LDs in Huh7 and LM3 cells using Oil red staining. Scale bar, 100 μm. **f**, **g** Wound-healing assay comparing the migration of Huh7 and LM3 cells. Scale bar, 500 μm. **h**, **i** Comparison of the migration and invasion of Huh7 and LM3 cells using transwell compartments. Scale bar, 400 μm. *P* values were determined by Student’s *t* test. *P < 0.05; **P < 0.01; ***P < 0.001.

To explore the mechanism by which SOCS5 promotes fatty acid biosynthesis, Huh7 cells were treated with LV-SOCS5 and 20 μM Fatostatin. Through targeted metabolomics of 39 medium- and long-chain FAs, when SOCS5 was overexpressed, multiple types of lipid synthesis became more active (concentration> 1 μg/10^7^ cells), and Fatostatin treatment reversed this effect (Fig. 6a; Supplementary Fig. 6h, i). Especially, the foldchange of seven FAs, including palmitate, stearate, cis-10-heptadecenoate, cis-11/14-eicosadienoic acid methyl ester, etc., was the most significant (Fig. 6b). Western blot experiments also showed that overexpression of SOCS5 promoted the expression of SREBP1, mSREBP1 and its downstream target genes, and 20 μM Fatostatin reversed this effect (Fig. 6c). Subsequently, the content of LDs in HCC cells was detected by oil red O staining. SOCS5 overexpression increased the content of LDs in Huh7 cells, while Fatostatin treatment reversed this effect (Fig. 6d). Similarly, SOCS5 knockdown alone reduces the lipid content in LM3 cells. The combined application of Fatostatin and SOCS5 knockdown significantly reduced the accumulation of intracellular LDs (Fig. 6e).

To further demonstrate whether SOCS5-SREBP1 mediated lipogenesis leaded to HCC invasion and migration, we found that overexpression of SOCS5 increased the invasion and migration capacity of Huh7 cells through wound healing and transwell experiments, while Fatostatin can reverse this effect (Fig. 6f, h). Similarly, the combination of Fatostatin and SOCS5 knockdown significantly enhanced the inhibition of migration and invasion of LM3 cells (Fig. 6g, i). These results suggest that SOCS5-SREBP1 induces lipid synthesis and promotes HCC migration and invasion.

### SREBP1 inhibition attenuates the carcinogenic effects of SOCS5 in vivo

**Fig. 7:**
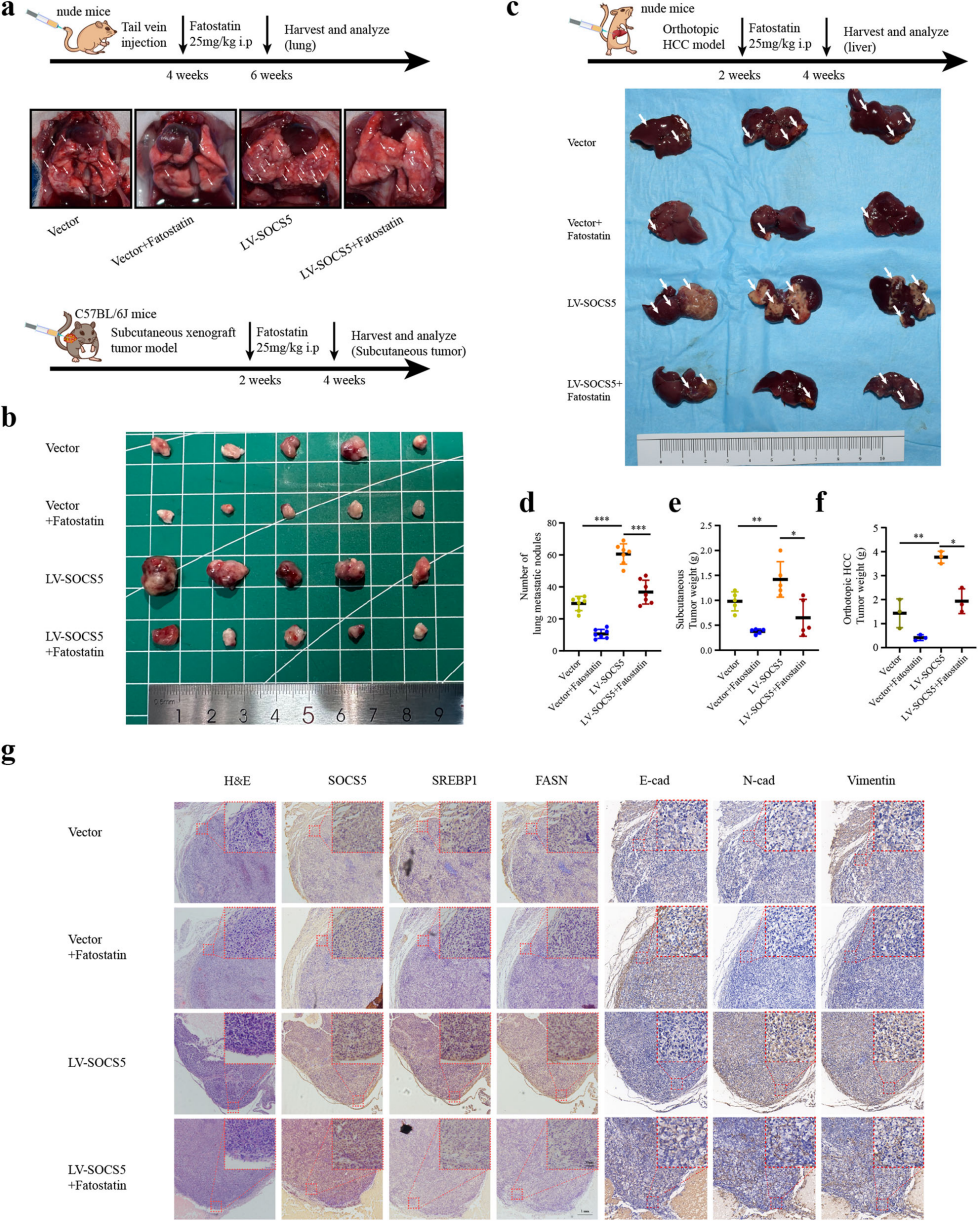
SREBP1 inhibition attenuates the carcinogenic effects of SOCS5 in vivo. **a**, **d** Images of lung tissues from nude mice, and the number of metastatic nodules in the lungs. **b**, **e** Images of subcutaneous tumor in C57BL/6 mice, and the tumor volume of four groups. **c**, **f** Images of liver orthotopic xenograft tumor from nude mice, and quantifications of liver tumor weight. *P* values were determined by Student’s *t* test. **g** HE staining and IHC staining of SOCS5, SREBP1, FASN, E-cadherin, N-cadherin, and Vimentin in liver orthotopic xenograft tumor. The results presented as mean ± SD. *P < 0.05; **P < 0.01; ***P < 0.001.

We further examined the in vivo SOCS5-SREBP1-mediated carcinogenic effects through tail vein–lung metastasis, liver orthotopic xenograft, and subcutaneous tumorigenesis models. The number of lung metastatic nodules in nude mice inoculated with Huh7^LV-SOCS5^ cells was significantly increased compared with the Huh7^Vector^ control group, indicating that the metastatic capacity of HCC Huh7 cells was significantly increased after SOCS5 overexpression. However, the lung metastatic nodules were significantly decreased after treatment with Fatostatin (Fig. 7a, d). Meanwhile, in order to better mimic the clinical scenarios, C57BL/6 mice with complete immunity were subcutaneously injected with Hepa1-6^LV-SOCS5^ cells and Hepa1-6^Vector^ cells. The subcutaneous tumor volume and weight in C57BL/6 mice inoculated with Hepa1-6^LV-SOCS5^ cells were significantly increased compared with the Hepa1-6^Vector^ control group, while the tumor volume and weight were significantly decreased after treatment with Fatostatin (Fig. 7b, e).

In addition, we further established a liver orthotopic xenograft nude mice model. Compared with that in the Huh7^Vector^ group, the number of tumor nodules and volume on the liver in the Huh7^LV-SOCS5^ group increased significantly, while the growth of tumors on the liver was significantly inhibited after treatment with Fatostatin (Fig. 7c, f). Importantly, IHC was used to evaluate the expression levels of SOCS5, SREBP1, FASN, E-Cadherin, N-Cadherin, and Vimentin in nude mice HCC. Compared with the vector group, the expression levels of SOCS5, SREBP1, FASN, N-Cadherin, and Vimentin in the LV-SOCS5 group were significantly enhanced, and the expression level of E-Cadherin was significantly decreased. The application of Fatostatin reduced the expression levels of SREBP1, FASN, N-Cadherin, and Vimentin, and increased the expression level of E-Cadherin, which inhibited the carcinogenic effect of SOCS5 (Fig. 7g).

## Discussion

**Fig. 8:**
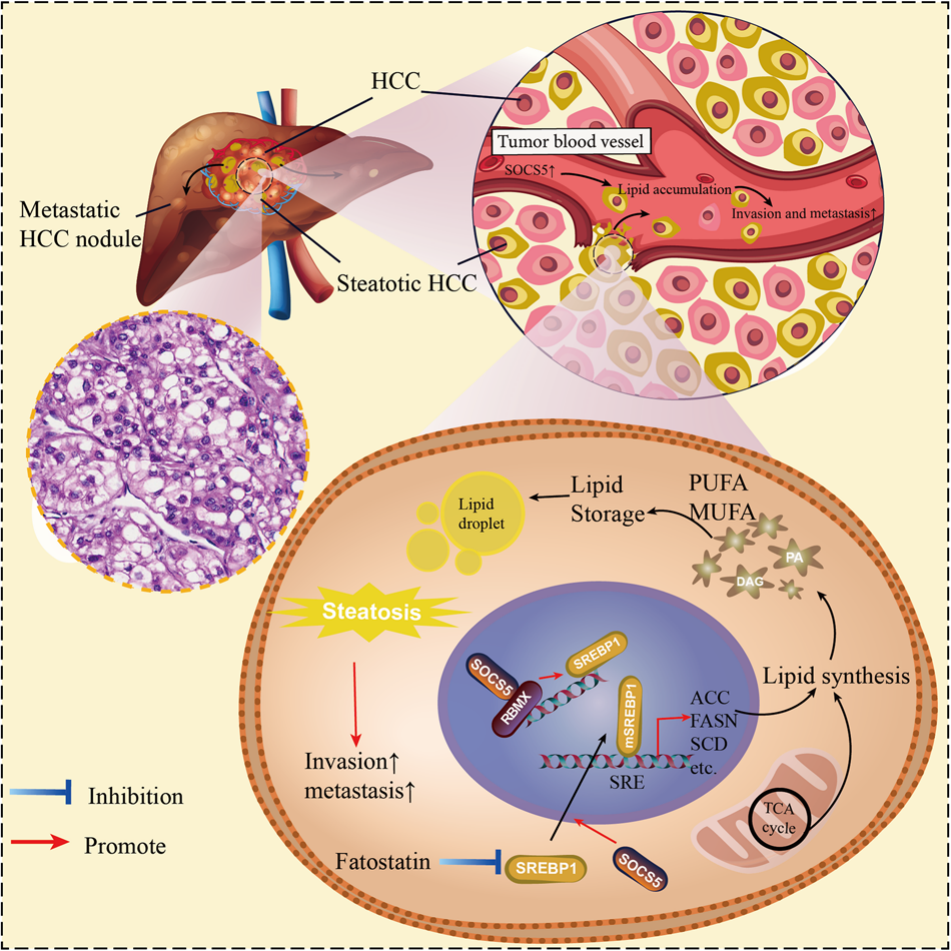
Schematic of SOCS5 promotes lipogenesis and metastasis in HCC. SOCS5 stimulates the promoter of SREBP1 by interacting with RBMX in the nucleus, inducing de novo lipogenesis to promote HCC metastasis.

The liver, as the main metabolic organ, mainly regulates the metabolic homeostasis^18^, while the occurrence and progression of HCC clearly breaks this balance^19,20^. HCC is considered a metabolism-related disease, and abnormal enhancement of FA synthesis promotes the metastasis and malignant progression of HCC^21^. This study described SBC-HCC as a special type of HCC, with a larger tumor volume and worse prognosis. We demonstrate that SOCS5 is one of the oncogenes of SBC-HCC associated with the ability to promote HCC metastasis by promoting de novo lipogenesis through the regulation of SREBP1. SOCS5-SH2 domain, especially the amino acids Y413 and D443, act as a critical binding site for the RBMX-RRM domain. SOCS5-RBMX costimulates the promoter of SREBP1, which enhances the transcription of key enzymes for lipid synthesis, while mutations in the SH2 domain, Y413, and D443 reverse this effect. This may be the cause of SBC-HCC steatosis and metastasis (Fig. 8).

Recent studies describe a similar pathological subtype, steatohepatitic HCC, characterized by partial HCC pathological exhibition of “steatohepatitis”. Salomao et al. described SH-HCC cases from New York State in 2012^11^. In terms of clinical features, the majority of SH-HCC patients in the study had fatty liver (93.7%) and HCV (43.7%) backgrounds. Subsequently, Shibahara reported cases of SH-HCC from the Tokyo area of Japan in 2014^10^. In terms of clinical features, 46.2% of SH-HCC cases in the Tokyo area had an HCV background. Clearly, HCC patients with a fatty liver background are often accompanied by abnormal lipid accumulation in the liver^22^. Similarly, HCV infection can induce insulin resistance and inhibit microsomal transfer protein activity, leading to the accumulation of triglycerides in the liver^23,24^. These factors may be directly involved in the development of SH-HCC. However, 70–90% of patients with chronic HBV infection develop liver fibrosis and cirrhosis rather than steatohepatitis, and the patterns of metabolic alterations are currently unclear^25^. Interestingly, Shibahara also reported 71 HCC cases with HBV( + )HCV(-), of which 13 cases (18.3%) were consistent with the diagnosis of SH-HCC, which seems to indicate that HBV-related HCC can also undergo abnormal lipid metabolism. However, the lack of fatty liver background in HCC patients in Shibahara’s study poses uncertain effects on the present conclusion. Therefore, it is necessary to further clarify this special HBV-related steatotic HCC subtype and explore its driver genes.

In China, approximately 85% of HCC cases develop from HBV-related cirrhosis^26^. We carried out a study involving a large number of Chinese HCC patients to probe the clinical features and drivers of HBV-related steatotic HCC in detail. In our HCC cohort, 88.1% of HCC patients had HBV-related cirrhosis. As shown in electron micrographs and pathological images, SBC-HCC patients were accompanied by intracellular LDs deposition in tumor cells, and their steatotic vacuoles only appeared in tumor tissues, but not in adjacent or distant tumor tissues. The SH-HCC cohorts of Salomao and Shibahara were usually accompanied by hypertriglyceridemia and hypercholesterolemia. In contrast, our SBC-HCC group did not show significant dyslipidemia compared to the non-SBC-HCC group, with only slightly higher serum TG and LDL-C levels (*P* < 0.05), but their expression was also within the normal range. This may be due to the lack of a fatty liver background in our patients, and the absence of steatotic vacuoles in adjacent normal tissues also supports this view. Intratumoral steatosis causes LDs deposition and increased tumor volume, which explains why SBC-HCC has a larger tumor size than non-SBC-HCC.

Zucman-Rossi previously classified HCC into 2 major classes: a proliferation class and a non-proliferation class. The proliferation class is associated with HBV infection and contains the TGFβ-Wnt subclass characterized by much higher aggressiveness, worse prognosis, immunodepletion and frequent activation of survival-related signaling pathways (cell cycle, mTOR, TGFβ-Wnt), accompanied by higher TP53 mutation rates, which is similar to our SBC-HCC^27^. Although the authors did not evaluate the intratumoral steatosis of the TGFβ-Wnt subclass in their study, the results suggest that our SBC-HCC may be closely related to the TGFβ-Wnt subclass. Interestingly, among the non-proliferation classes associated with HCV infection and alcohol, Boyault’s G4 subclass and Murai’s Class II subclass are characterized by steatohepatic HCC, a well differentiated tumor and good prognosis, with an active immune response, and lower TP53 mutation rates^28–30^. This is similar to the steatotic HCC subtype we identified in the TCGA HCC cohort. These data show the unique mutation, transcriptomics, and pathological features of SBC-HCC in the Chinese population, which differ from HCV and other nonviral steatotic HCC cohorts (Supplementary Fig. 2). Identifying the driver genes that cause intracellular steatosis of HBV-related HCC may serve as a therapeutic target for SBC-HCC.

In the Gao’s HBV-related cirrhosis HCC cohort and GSE121248’s HBV-related HCC cohort, we found that high expression of SOCS5 predicts increased FA synthesis, and SOCS5, as one of the driver genes for SBC-HCC, is upregulated in SBC-HCC. De novo lipogenesis is known to be the maintenance of energy supply and as a pro-tumor signaling molecule, which is essential for tumorigenesis and malignant progression^31^. However, more complex mechanisms of the carcinogenic effects of FAs remain to be explored. In addition to maintaining membrane structure and fluidity, the increased de novo lipogenesis in tumor cells leads to saturated phospholipid production, which may increase membrane stiffness and avoid ROS-induced damage^32^. Some FAs also lead to immunosuppressive phenotypes by promoting PD-L1 expression^29^. In this study, we demonstrate that the high expression of SOCS5 promotes SREBP1-mediated de novo lipogenesis and LD production and is one of the driver genes for HCC lipid metabolism reprogramming. The family of SREBPs includes SREBP1 and SREBP2, both of which bind to steroid regulatory elements (SREs) but prefer different promoters and have tissue-specific expression. SREBP1 primarily regulates genes associated with FA synthesis and is primarily expressed in the liver, while SREBP2 is primarily involved in cholesterol biosynthesis pathways^33,34^. In addition, Thomas et al. previously demonstrated that mTOR1 is required for SREBP1 activation (mSREBP1) and that rapamycin inhibits the mTOR-Akt-induced nuclear accumulation of mSREBP1^35^. We also found that SOCS5 promotes the nuclear transfer of SREBP1, which may be related to our previous discovery that SOCS5 activates the PI3K/AKT/mTOR pathway^12^. These results contribute to a deeper understanding of the molecular mechanisms by which SOCS5 promotes HCC steatosis and metastasis, and our study fills this gap.

To further explore the mechanisms of SOCS5, we demonstrated the direct binding of SOCS5 and RBMX both in vivo and in vitro through co-IP and GST-pulldown experiments. RBMX is an RNA-binding protein that plays several roles in the regulation of pre- and post-transcriptional processes^36^. RBMX has been shown to be one of the oncogenes for HCC, is highly expressed in HCC and predicts a poor prognosis^13^. In addition, Takemoto et al. confirmed with two independent methods that RBMX binds to the region between −453 and −480 bp of SREBP1 promoter, and increases the pre-transcriptional activity of the SREBP1 promoter^14^. We found that the protein expression levels of SOCS5 and RBMX were positively correlated, revealing a combined practical value for predicting OS in HCC patients. We demonstrated that overexpression of SOCS5 increased the protein stability of RBMX and increased the accumulation of RBMX within the nucleus. In fact, there is no evidence of the presence of DNA-binding domains in either SOCS5 or RBMX proteins. We speculate that within the nucleus, SOCS5-RBMX may form a complex with another DNA-binding protein that costimulates the activity of the SREBP1 DNA promoter. More importantly, structural analysis and co-IP experiments found that the amino acids Y413 and D443 in the SOCS5-SH2 domain are the critical sites for direct binding to the RBMX-RRM domain. Mutations in the SH2 domain, Y413, and D443 reverse the activation effects of the SOCS5-RBMX complex on the SREBP1 promoter. These results lay the foundation for the design of a specific blocking drug for SOCS5-RBMX to target abnormal lipid metabolism for SBC-HCC treatment.

In summary, this study precisely identified that SBC-HCC is a special HCC subtype and screened SOCS5, an oncogene for FA synthesis of HCC that is upregulated in SBC-HCC. Mechanically, we demonstrate that SOCS5-SH2 domain, especially the amino acids Y413 and D443, act as a critical binding site for the RBMX-RRM domain. We found that SOCS5 stimulates the promoter of SREBP1 by interacting with RBMX, inducing de novo lipogenesis to promote HCC metastasis, while mutations in the SH2 domain, Y413, and D443 reverse this effect. The development of specific inhibitors targeting the critical binding sites of SOCS5-RBMX could provide targeted therapeutic effects on SBC-HCC with fewer adverse effects.

## Materials and methods

### Patient samples

This study was approved by the Institutional Review Board of the Affiliated Hospital of Qingdao University (QYFYWZLL27315). All patients provided written informed consent to participate. From January 2013 to December 2016, 245 consecutive HCC patients from the Affiliated Hospital of Qingdao University were recruited. The inclusion criteria were as follows: age ≥18 years; Child‒Pugh grade A; primary surgical resection of HCC; pathologically confirmed HCC, including single or multiple tumors; and no hilar lymph node involvement or extrahepatic metastasis. The methodology and design of this study strictly adhered to the ethical guidelines of the Helsinki Declaration of 1975 (revised in 2013). The Ethics Committee of the Affiliated Hospital of Qingdao University approved the study. The protein expression level of SOCS5 in 245 paraffin-embedded HCC tissues was examined by IHC. An additional 16 frozen HCC tissues and matched adjacent non-tumor liver tissues were collected from patients who underwent surgery at the Affiliated Hospital of Qingdao University in 2022.

The diagnosis of SBC-HCC was made if the tumor fulfilled the following five criteria: intratumoral steatosis (>5% tumor cells), no steatosis in adjacent normal tissues, HBV infection, HBV-related cirrhosis, and no background of fatty liver. Patients with hepatitis B surface antigen were identified as HBV (+). Liver cirrhosis was diagnosed based on pathologic findings. In our cohort of 245 HCC patients, 43 had steatosis (>5% tumor cells), of which 41 were diagnosed with SBC-HCC. Other 2 cases had no HBV infection and cirrhosis, and no fatty liver background cases. In addition, the diagnostic criteria for non-SBC-HCC (*n* = 175) were no steatosis in tumor and adjacent normal tissues, HBV infection, HBV-related cirrhosis, and no background of fatty liver.

### Cell culture

HCC Huh7 cell line and HEK 293T cell line (contain STR profiling, without mycoplasma contamination) were purchased from the Procell Life Science & Technology (Wuhan, China). HCC cell lines (HCCLM3 and PLC/PRF/5, containing STR profiling, without mycoplasma contamination) were purchased from a cell bank at the Chinese Academy of Sciences (Shanghai, China).

Authentication of HCC cell lines used in this study was performed by short tandem repeat (STR) DNA Profiling in June 2020 and no cellular cross-contamination was detected. All cells were cultured in Dulbecco’s modified Eagle’s medium (DMEM, Hyclone, USA) supplemented with 10% FBS (Gibco, USA) and 1% P/S (Gibco, USA), and incubated at 37 °C in a 5% CO_2_ incubator.

### Transfections and cell treatments

All siRNA and plasmid were purchased from GenePharma (Shanghai, China) and Genomeditech (Shanghai, China), respectively. Lipofectamine 3000 (Invitrogen) was used to transfect the siRNA and plasmid. Lentivirus of short hairpin RNAs (shRNAs) targeting SOCS5, SOCS5 overexpression lentivirus and the control lentivirus were purchased from Genomeditech (Shanghai, China). After lentivirus infection of Huh7 and HCCLM3 cells (MOI = 10), stably infected cell lines were screened using puromycin (MedChemexpress) according to the instructions. Cells were treated with 10–40 μM Fatostatin (MedChemexpress) for 24 h to Inhibit SREBP1. siSOCS5#1(5′–3′): AACCAGUCAAGGCAAAGUATT; siSOCS5#2(5′–3′): GAACCAGUCAAGGCAAAGUTT; siRBMX#1(5′–3′): CGGAUAUGGUGGAAGUCGATT; siRBMX#2(5′–3′): UCAAGAGGAUAUAGCGAUATT; shSOCS5(5′–3′): AACCAGTCAAGGCAAAGTA/TACTTTGCCTTGACTGGTT.

### Antibodies and reagents

SOCS5 (1:500, sc-100858, Santa Cruz); HA-Tag (1:1000, #3724, CST); hnRNP G (1:1000, ab190352, Abcam); FASN (1:1000, #3180, CST); Myc-Tag (1:1000, #2276, CST); ACC (1:1000, #3676, CST); SCD1 (1:1000, ab236868, Abcam); ACLY (1:5000, ab40793, Abcam); SREBP1 (1:1000, 14088-1-AP, Proteintech); IgG (1:1000, #2729, CST); HRP anti-mouse IgG (1:10,000, abs20001, Absin); HRP anti-rabbit IgG (1:10,000, abs20002, Absin); CD36 (1:1000, ab252922, Abcam); FATP4 (1:1000, ab200353, Abcam).

### Oil red O staining

Tissue samples from HCC patient and HCC cell lines were stained with oil red kit (G1261, Solarbio). Wash by water after fix in 10% formalin for 10 min. Rinse sections slightly with distilled water. Soak sections in 60% isopropanol for 20–30 s. Stain section in Modified Oil Red O Stain Solution (capped) for 10–15 min. Color separation: Wash slightly in 60% isopropanol to remove the dye solution. Re-dyeing by Mayer’s Hematoxylin Solution for 1–2 min. Slightly differentiate by 1% hydrochloric acid solution. Rinse sections slightly with distilled water.

### Immunofluorescence

Immunofluorescence staining was performed on cells cultured on coverslips. Cells were fixed in 4% paraformaldehyde for 20 min. Intracellular epitope detection was performed in cells permeabilized in 0.1% Triton X-100 in PBS for 10 min. Following blocking with 5% bovine serum albumin (BSA) for 30 min, cells were exposed overnight at 4 °C to SOCS5 (1:50, sc-100858, Santa Cruz), RBMX (1:100, ab190352, Abcam) and SREBP1 (1:50, 14088-1-AP, Proteintech) antibodies. Primary antibodies were revealed using anti-rabbit IgG (1:500, #4413, CST) or anti-mouse IgG (1:100, abs20003, Absin) secondary antibodies. 4′,6-Diamidino-2-phenylindole (DAPI) was used to counterstain the nuclei.

### Immunoprecipitation (IP) assay

The cells were suspended in NP-40 lysis buffer (Beyotime, P0013F) for 30 min on ice and centrifuged at 12,000 × *g* for 10 min. Suspension Protein A/G Magnetic Beads (MedChemExpress, HK-K0202), Anti-HA Magnetic Beads (MedChemExpress, HK-K0201) or Anti-c-Myc Magnetic Beads (MedChemExpress, HK-K0206): Take 10 μL Magnetic Beads and put them in 1.5 mL EP tubes. Add 500 μL washing buffer and suspend them fully. Place them in Magnetic racks for Magnetic separation and discard supernatant. Repeat the washing procedure 2 times. IgG antibodies were diluted to a final concentration of 25 μg/mL using binding/washing buffer. The diluted 400 μL IgG antibody was added to the treated magnetic beads and incubated in a flipping mixer (room temperature, 30 min; 4 °C, 2 h), magnetic minutes Off, collect the magnetic beads. 500 μL cell lysate was added to the magnetic beads and incubated in a turnover mixer, overnight at 4 °C. Magnetic beads were washed 3 times with washing buffer. Magnetic beads were added with 50 μl of 1× protein loading buffer and heated at 95 °C for 5 min. After cooling, magnetic separation was performed, and the supernatant was removed for Western blot analysis.

### Measurement of free fatty acids (FFAs)

The cells were collected, and subsequent experiments were performed according to the protocol of the Free Fatty Acid Quantitation Kit (MAK044, Sigma). Cells (1 × 10^6^) can be homogenized in 200 μL of a 1% (w/v) Triton X-100 in chloroform solution. Centrifuge the samples at 13,000 × *g* for 10 min to remove insoluble material. Collect the organic phases (lower phase) and air dry at RT remove chloroform. Vacuum dry for 30 min to remove trace chloroform. Dissolve the dried lipids in 200 μL of Fatty Acid Assay Buffer by vortexing extensively for 5 min. Bring samples to a final volume of 50 μL with Fatty Acid Assay Buffer. Add 2 μL of ACS Reagent to each sample and standard well, and incubate for 30 min at 37 °C. Set up the Master Reaction Mix (50 μL) of the Master Reaction Mix is required for each reaction (well). Add 50 μL of the Master Reaction Mix to each of the wells. Mix well using a horizontal shaker or by pipetting, and incubate the reaction for 30 min at 37 °C. Protect the plate from light during the incubation. For colorimetric assays, measure the absorbance at 570 nm. For fluorometric assays, measure fluorescence intensity.

### Protein–protein docking prediction and molecular dynamics simulation

The 3D structural models predicted by AlphaFold of SOCS5 and RBMX were obtained (https://www.uniprot.org/). ZDOCK 3.0.2 is used for protein docking prediction (https://zdock.umassmed.edu/). The complex diagram was visualized with PyMOL 2.2.0.

The Simulation and Standard Dynamics Cascade modules of Discovery Studio 2019 software were used to obtain the position parameters. The molecular parameters of the ligand during the simulation process were adopted by the Charmm36 field, and the molecular parameters of the acceptor protein were adopted by the Charmm36 field. Solvation of protein-ligand complexes during Solvation module calculations. Then run molecular dynamics simulations, including 5 stages: I Minimization, I Minimization2, Heating, Equilibration, and Production. View conformational change animations that show changes during dynamic simulations.

### Immunohistochemical (IHC) analysis

IHC staining was done on 245 paraffin-embedded HCC tissues collected at the Affiliated Hospital of Qingdao University between January 2013 to December 2016 to determine the level of SOCS5 protein expression. 115 paraffin-embedded HCC tissues collected at the Affiliated Hospital of Qingdao University between January 2015 to December 2016 to determine the level of RBMX protein expression. On the basis of the brightness of staining (0 = negative, 1 = weak, 2 = medium, or 3 = strong) and the percentage of strongly stained cells within the observed field (0 = 0%, 1 = 1–25%, 2 = 26–50%, and 3 ≥ 51%), scoring of IHC staining were multiplied by brightness and percentage scores. A final score of < 4 was defined as low expression and a score of 4–9 was defined as high expression. Chi-square tests were used to determine the correlations between SOCS5 expression levels and the clinicopathological characteristics of HCC patients.

### Dual-luciferase reporter assay

Cells were transfected with plasmids expressing SREBP1 (MCS-firefly_Luciferase-SREBP1, Genechem, Shanghai, China), plasmids expressing empty vector (MCS-firefly_Luciferase-Vector, Genechem, Shanghai, China), Renilla plasmids (promoter-Renilla_Luciferase, Genechem, Shanghai, China). The treated cells were collected and the Luciferase expression of SREBP1 was detected by Dual Luciferase Reporter Assay Kit (DL101-01, Vazyme). Discard cell culture medium, wash twice with PBS, and add 100 μL 1 × Cell Lysis Buffer/well to 24-well. Lyse at RT for 5 min and pipette the cell lysate into a 1.5 ml EP tube. Centrifuge 12,000 × *g* at RT for 2 min and take the supernatant for subsequent detection. Add 100 µl of RT Luciferase substrate to the microtiter plate, carefully pipette 20 µl of the cell lysate supernatant into the wells of the microtiter plate, mix quickly, and immediately detect the activity of the Firefly luciferase reporter gene in the microplate reader. Add 100 μl of freshly prepared Renilla substrate working solution to the above reaction solution, mix quickly, and immediately detect the activity of Renilla luciferase reporter gene in a microplate reader.

### Animals experiment

The treatment of mice was carried out in strict accordance with the principles approved by the Animal Experimental Ethics Committee of the Affiliated Hospital of Qingdao University. Twenty-eight male BALB/nude mice (4 weeks old) were purchased from Beijing Vital River Laboratory Animal Technology (Beijing, China) and randomly divided into two groups. HCC cells (2 × 10^6^) were suspended in 200 μL PBS. Mice were injected with 2 × 10^6^ Huh7^Vector^ cells or 2 × 10^6^ Huh7^LV-SOCS5^ cells through the tail veins. After 4 weeks, Fatostatin (25 mg/kg/bodyweight) was injected intraperitoneally three times a week^37^. After 8 weeks, all mice were anesthetized with CO_2_ and sacrificed by cervical dislocation, and the lungs were excised.

Twenty male C57BL/6 J mice (4 weeks old) randomly divided into two groups. HCC cells (3 × 10^6^) were suspended in 100 μL PBS. Ectopic HCC mouse models were established by subcutaneous injection of 3 × 10^6^ hepa1-6^Vector^ cells or hepa1-6^LV-SOCS5^ into left axilla of C57BL/6J mice. After 2 weeks, Fatostatin (25 mg/kg/bodyweight) was injected intraperitoneally three times a week. Once the tumors were palpable (tumor diameterå 1 cm), or after 6 weeks, all mice were anesthetized with CO_2_ and sacrificed by cervical dislocation, and the subcutaneous tumors were excised.

Twenty-four male BALB/C nude mice (4 weeks old) were randomly divided into two groups. Huh7^Vector^ cells or 2 × 10^6^ Huh7^LV-SOCS5^ cells (2 × 10^6^) were suspended in 40 μL serum-free DMEM/Matrigel (1:1) and orthotopically injected into the left hepatic lobe of 5-week-old male BALB/c nude mice. Two weeks later, there were 8 surviving mice in the Huh7^LV-SOCS5^ group and 9 surviving mice in the Huh7^Vector^ group. Subsequently, Huh7^LV-SOCS5^ group and Huh7^Vector^ group were separately and randomly divided into four groups: Vector, Vector+fatostatin, LV-SOCS5, and LV-SOCS5+fatostatin. Fatostatin (25 mg/kg/bodyweight) was injected intraperitoneally three times a week. Vector mice were injected only with the same volume of solvent liquid. Intraperitoneal injections lasted for four weeks. After 6 weeks, all mice were anesthetized with CO_2_ and sacrificed by cervical dislocation. The liver tissues were excised, and embedded in paraffin for hematoxylin and eosin staining or IHC staining.

### Statistical analysis

All experiments were repeated at least three times. SPSS 16.0 and GraphPad 8.0 were used for statistical analysis. Categorical variables were expressed as numbers (%) and tested by the χ^2^ test, continuity correction by χ^2^ test, or Fisher’s exact test. The results are expressed as the mean ± standard deviation (SD). Comparisons between two groups of continuous variable data were subjected to Student’s *t* test or the Wilcoxon test. Survival analysis was performed using Kaplan‒Meier method and the log-rank test. The two-variable correlation analysis was performed via the Pearson method. *p* < 0.05 was considered to indicate statistical significance. All western blotting, qPCR, dual-luciferase reporter assay and other experiments were repeated at least 3 times from distinct samples. *P < 0.05; **P < 0.01; ***P < 0.001.

### Other basic experiments

Details of lentivirus construction and infection of cell lines, RNA extraction and quantitative real-time PCR, western blotting analysis, cell migration, and invasion assays, wound-healing assay, and electron microscopy are presented in our previous study^12^. All blots derive from the same experiment and were processed in parallel. The PCR primers can be found in the Supplementary Information.

Un-cropped images of all blots are provided as Supplementary Figs. 9–14 in the Supplementary information file.

### Reporting summary

Further information on research design is available in the Nature Research Reporting Summary linked to this article.

### Supplementary information

Supplementary Information

Reporting Summary

## Data Availability

TCGA HCC RNA-seq data and diagnostic slides of 374 HCC patients were downloaded from TCGA dataset (https://portal.gdc.cancer.gov/), as well as clinical data on patient age, survival time, tumor staging, etc. In addition, RNA-Seq gene expression profiles of Gao’s HCC cohort (https://www.biosino.org/node/project/detail/OEP000321). RNA-Seq gene expression profiles of HBV-related HCC cohort from GSE121248 (https://www.ncbi.nlm.nih.gov). IHC images were obtained with the ProteinAtlas (https://www.proteinatlas.org/). The data of proteomics data generated in this study can be viewed in the Integrated Proteome Resources (https://www.iprox.cn/) database (accession no. PXD048740). All relevant data are available from the authors upon request. Supplementary Tables 5–9 in this study can be viewed in the Science Data Bank (https://cstr.cn/31253.11.sciencedb.15798. CSTR:31253.11.sciencedb.15798).

## Code availability

The R codes (R version 4.2.1) for the statistical analyses, and custom code or scripts in the generation or analysis of datasets are available from the corresponding author upon request.
