# Supplementary material for: SOCS5-RBMX stimulates SREBP1-mediated lipogenesis to promote metastasis in steatotic HCC with HBV-related cirrhosis
Source: NPJ Precis Oncol. 2024 Mar 1;8:58. doi: 10.1038/s41698-024-00545-6 (PMC10907597; doi:10.1038/s41698-024-00545-6)
Supplement: Supplementary file 1 — Supplementary Information [file 41698_2024_545_MOESM1_ESM.pdf]

Supplementary Information for:

"SOCS5-RBMX stimulates SREBP1-mediated lipogenesis to promote metastasis in steatotic HCC with HBV-related cirrhosis"

Youpeng Wang[1†], Ziyin Zhao[4†], Tingting Guo[1†], Tiansong Wu[1], Mao Zhang[2], Dingan Luo[1], Kunpeng Dou[3], Yeni Yang[1], Cheng Jin[5], Bingyuan Zhang[1], Bin Zhang[4*], Bing Han[1*]

[1]Department of Hepatobiliary and Pancreatic Surgery, Affiliated Hospital of Qingdao University, Qingdao, China;

[2]Liver Cancer Institute, Zhongshan Hospital, Fudan University, Shanghai, China.

[3]College of Information Science and Engineering, Ocean University of China, Qingdao, China.

[4]Organ Transplantation Center, The Affiliated Hospital of Qingdao University, Qingdao, Shandong, China.

[5]Institute of Medical Robotics, School of Biomedical Engineering, Shanghai Jiao Tong University, Shanghai, China.

This PDF file includes:

1. Supplementary Figures 1 to 14
2. Supplementary Tables 1 to 4
3. Supplementary methods

1. Supplementary Figures

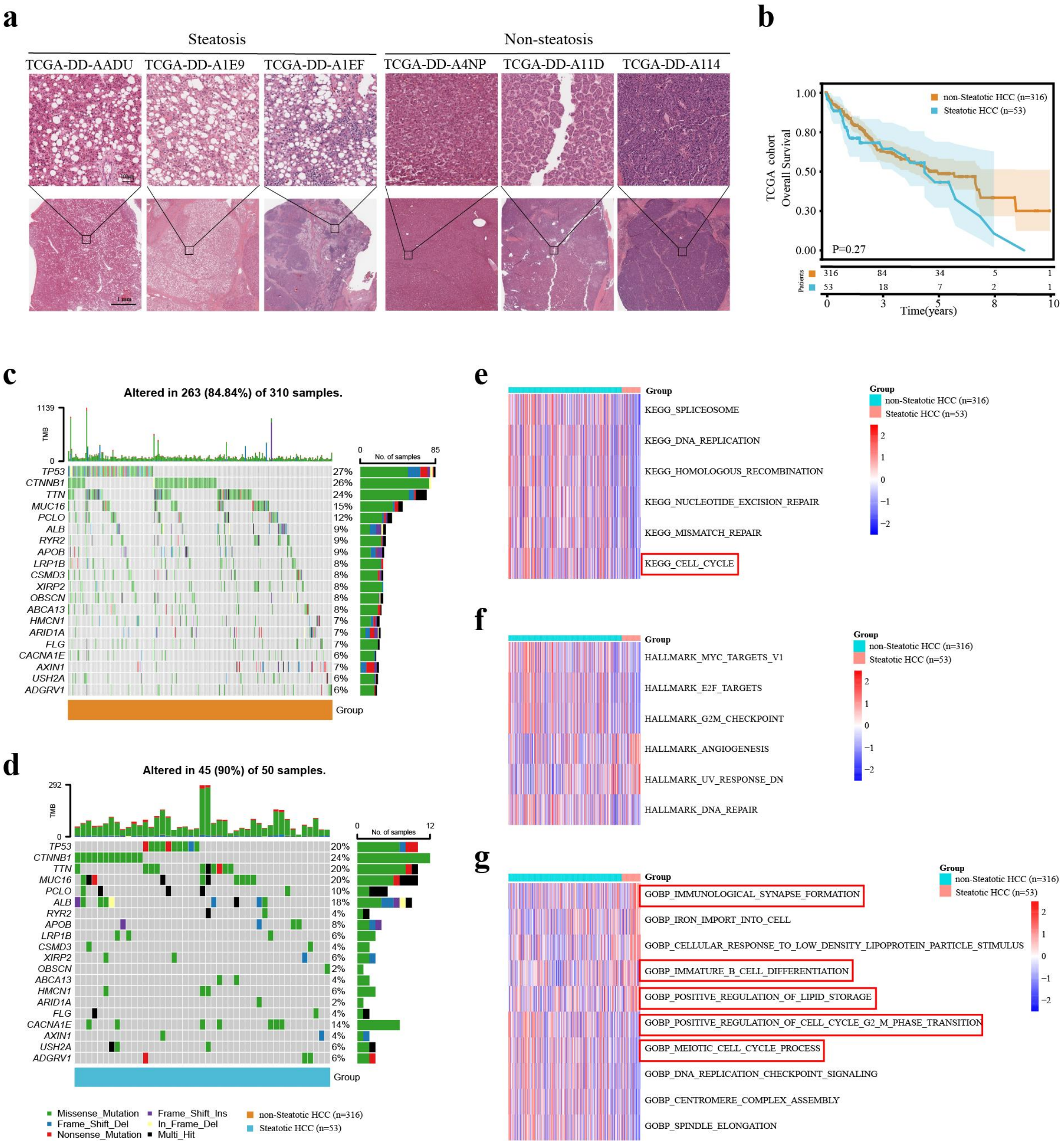

Supplementary Figure 1. Integration of HCC histological features, classifications and genetic alterations.

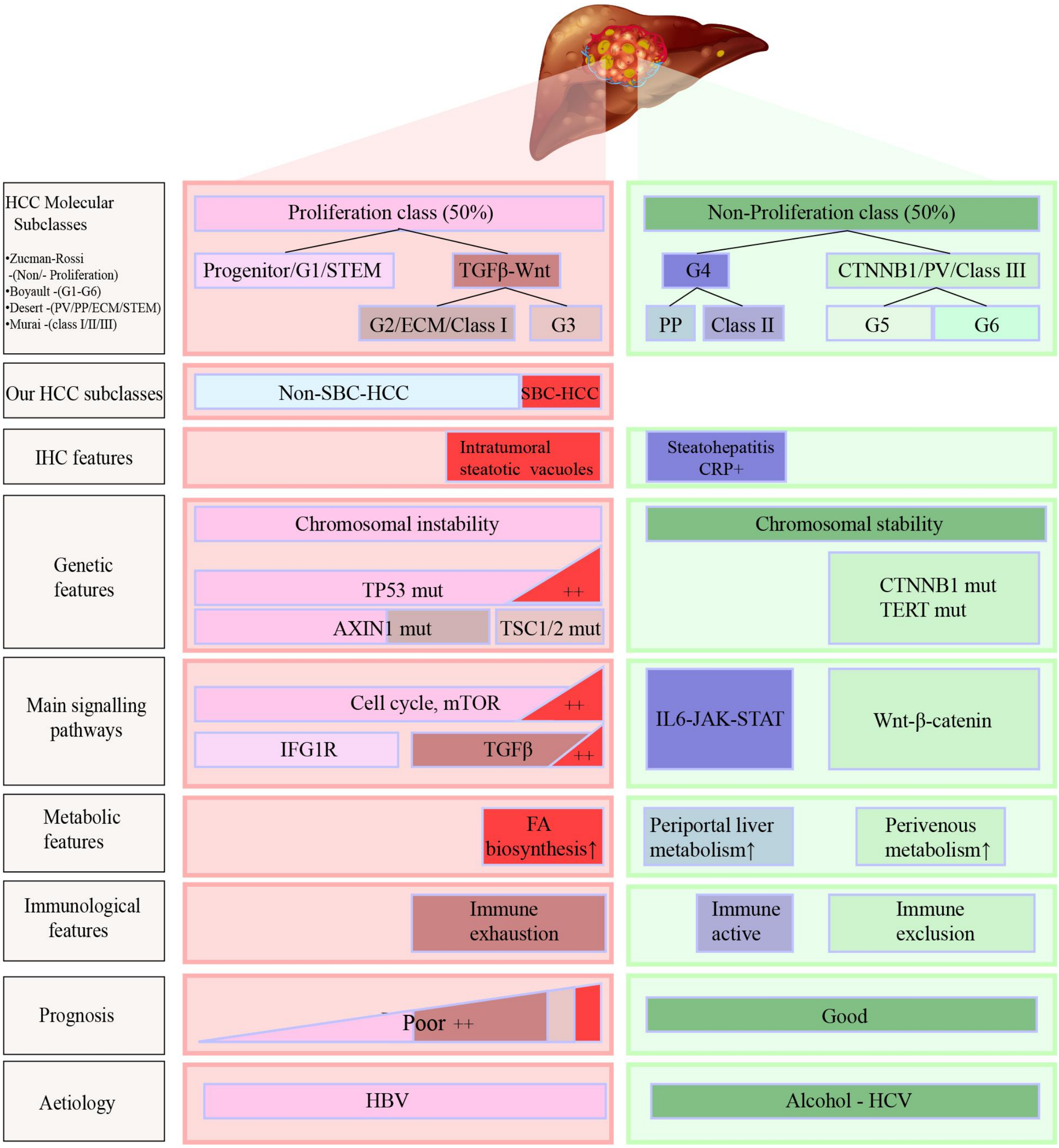

Supplementary Figure 2. Steatosis HCC and its characteristics in the TCGA dataset. (a) Tissue slide image for HCC patients with steatosis and non-steatosis in the TCGA database. (b) Kaplan-Meier overall survival of the steatosis group (n=53) and the non-steatosis group (n=316). P values were determined by the log-rank test. (c-d) Significantly mutated genes in the steatosis and the non-steatosis groups. (e-g) GSVA scores of patients in the steatosis and the non-steatosis groups. Log-rank

(Mantel-Cox) test was used for survival analysis.

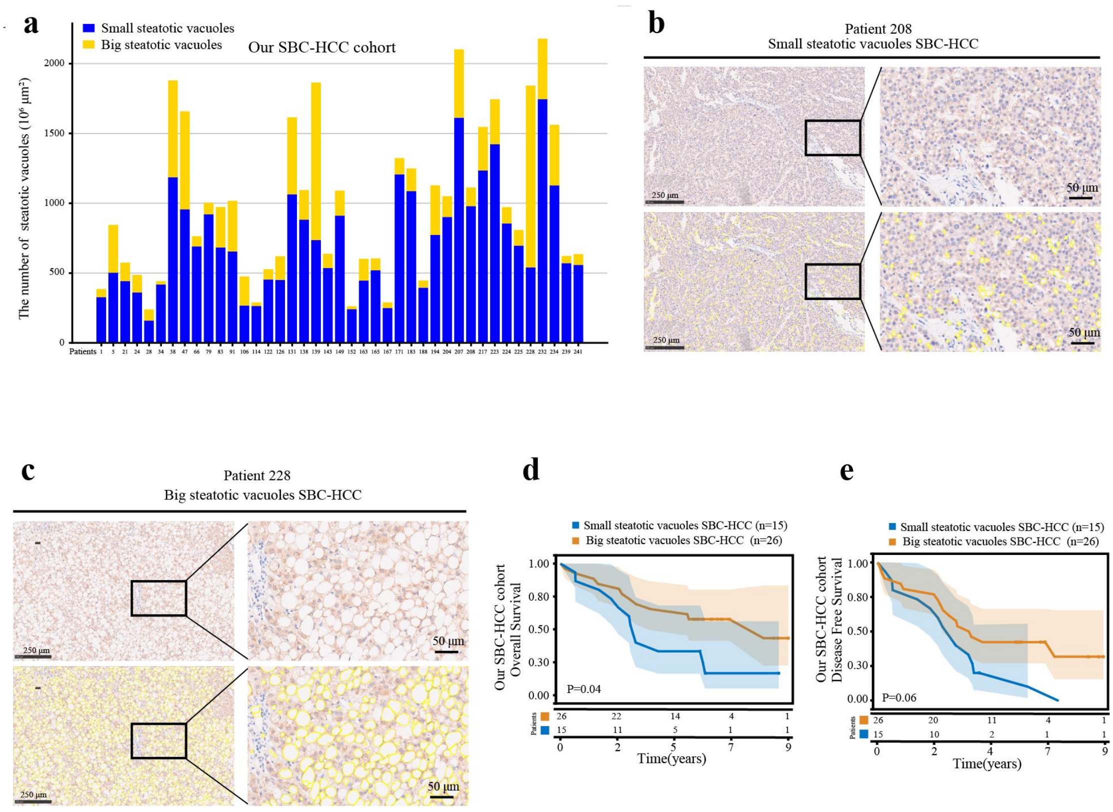

Supplementary Figure 3. Steatotic vacuoles characteristics of our SBC-HCC cohort. (a) The number of steatotic vacuoles in our SBC-HCC cohort (10^6 μm^2), > 82.5 μm^2 defined as big steatotic vacuoles, < 82.5 μm^2 defined as small steatotic vacuoles. (b-c) According to the optimal cut-off value, the proportion of small vacuoles > 86% defined as small steatotic vacuoles SBC-HCC (n=15), and < 86% defined as big steatotic vacuoles SBC-HCC (n=26). Representative images of the big and small steatotic vacuoles SBC-HCC. Scale bar, 250μm and 50μm. (d-e) Kaplan-Meier overall survival of the big and small steatotic vacuoles SBC-HCC. P values were determined by the log-rank test.

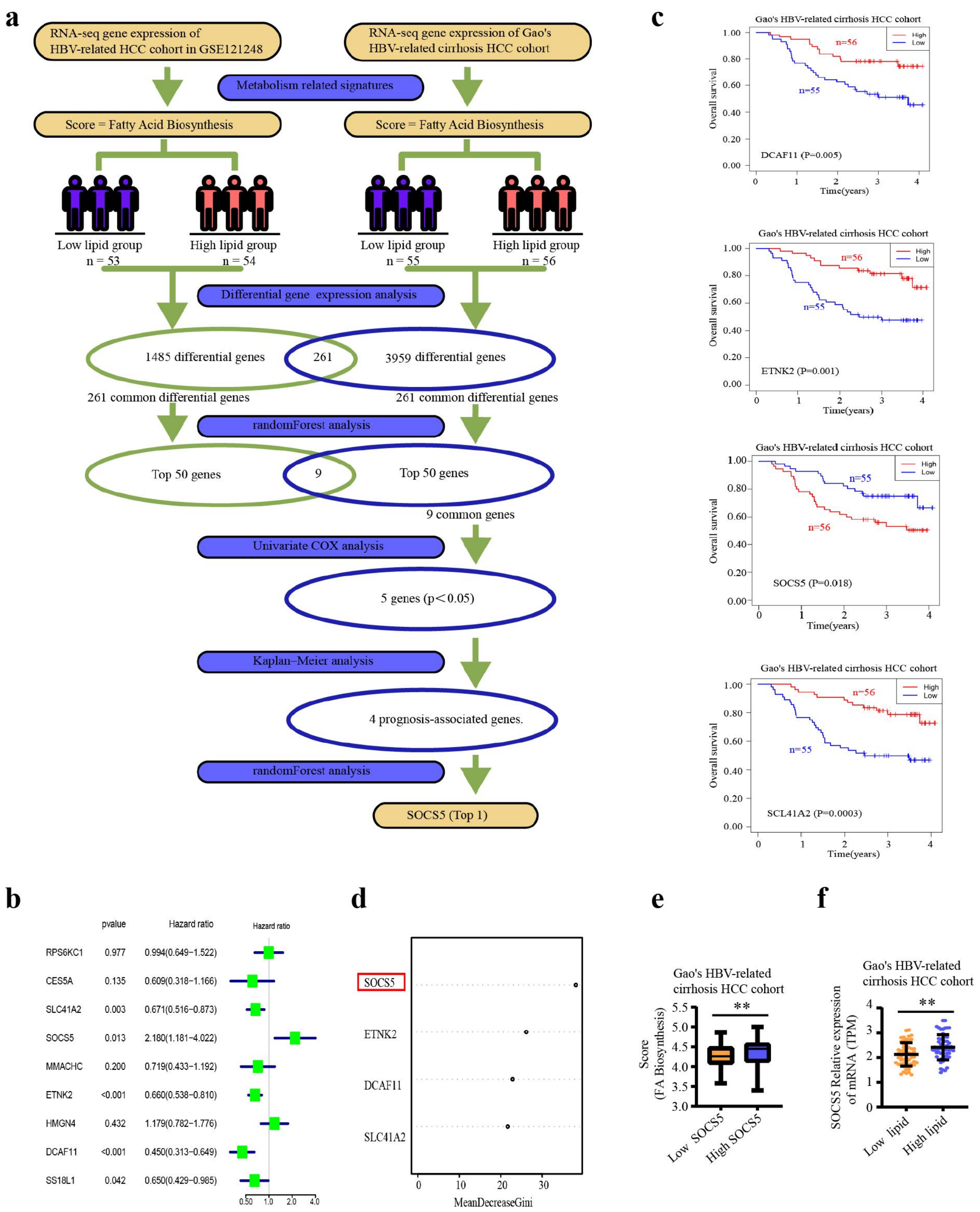

Supplementary Figure 4. Bioinformatics screening process for SBC-HCC driver gene SOCS5. (a) Flow chart of the screening of driver genes in Gao'HBV-related cirrhosis HCC cohort and GSE121248. (b) Forest plot of nine key genes. (c) Four key prognosis-associated genes in Gao'HBV-related cirrhosis HCC cohort. P values were determined by the log-rank test. (d) SOCS5 is the most critical disease signature gene

in the random forest model (ntree=1000). (e-f) In Gao' HBV-related cirrhosis HCC cohort, the expression of SOCS5 in the High Lipid group (n=56) was upregulated, and SOCS5 high expression group suggested higher score of FA Biosynthesis. P values were determined by Student's t test. *P < 0.05; **P < 0,01; ***P < 0.001.

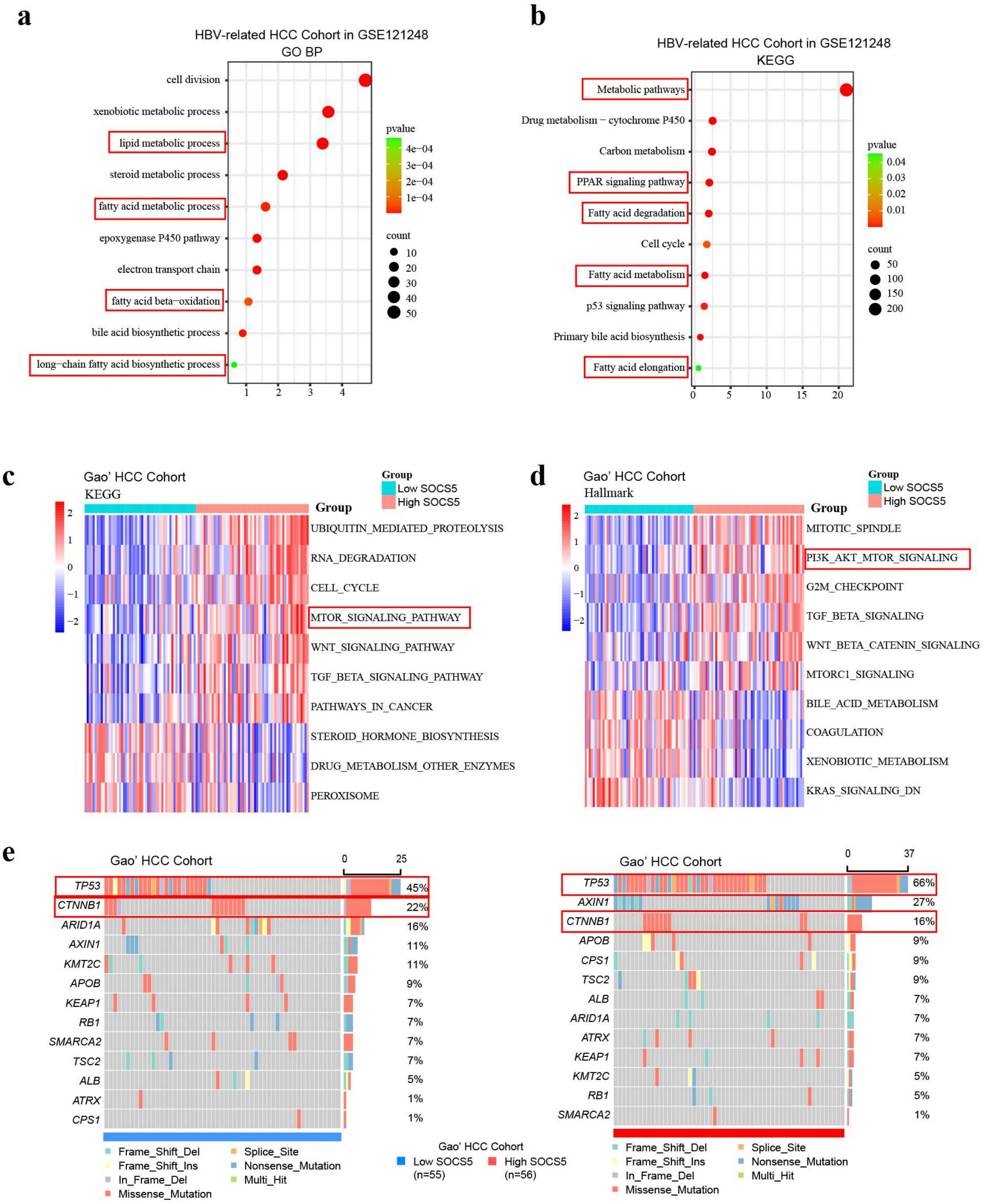

Supplementary Figure 5. Public databases show that SOCS5 is closely related to

lipid metabolism. (a-b) In the GSE121248, GO and KEGG functional enrichment analysis of differential genes between the Low and High SOCS5 patients. (c-d) GSVA scores of patients in the high SOCS5 and low SOCS5 groups in Gao' HBV-related cirrhosis HCC cohort. (e) Significantly mutated genes in high SOCS5 and low SOCS5 groups in Gao' HBV-related cirrhosis HCC cohort.

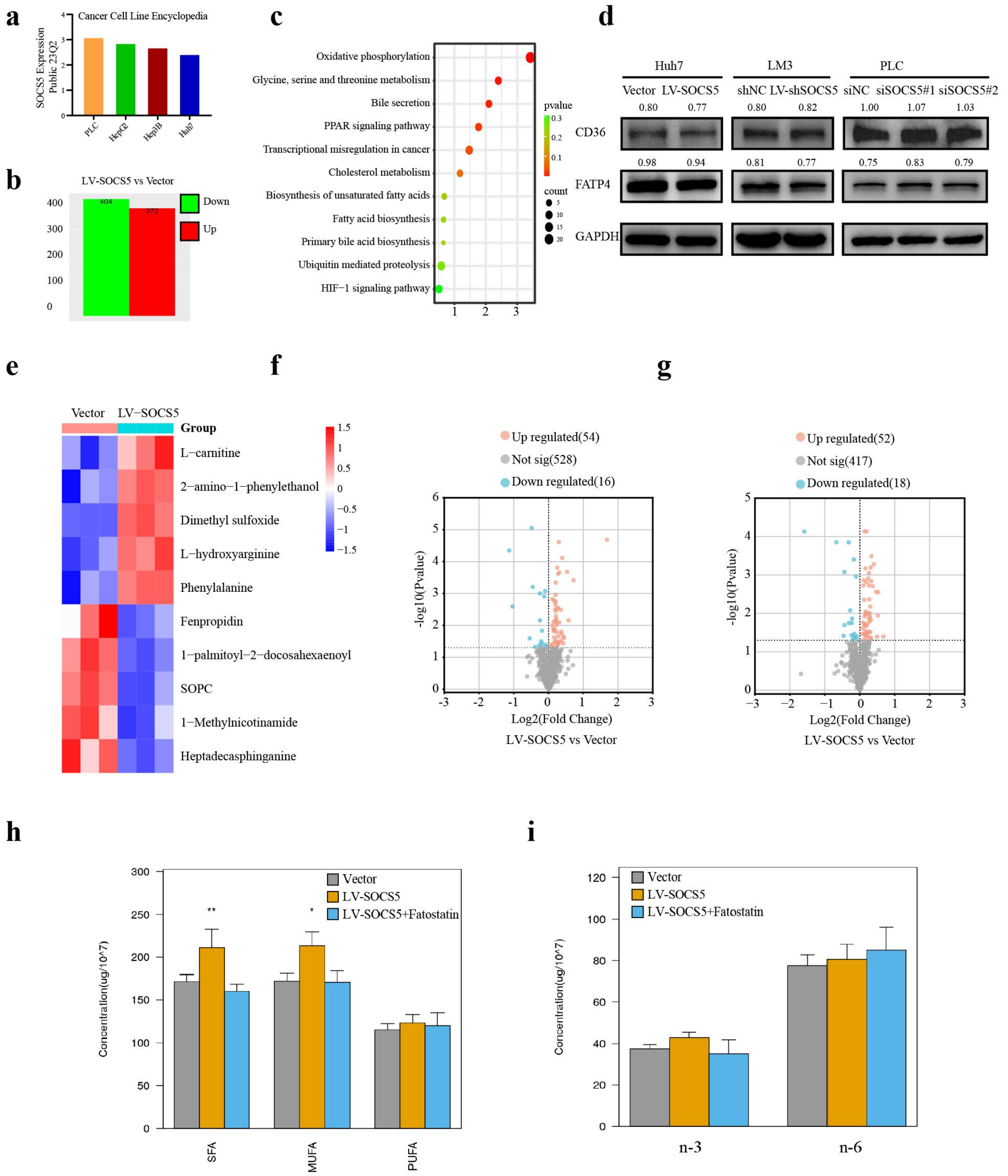

Supplementary Figure 6. Metabolomics and proteomics reveal that SOCS5 is closely related to lipid metabolism. (a) The relative expression levels of SOCS5 in Hep3B, HepG2, Huh7, and PLC in CCLE database. (b) After overexpression of SOCS5 in Huh7 cells, proteomics identified 372 upregulated proteins and 404 downregulated proteins ($P<0.05$). (c) KEGG Enrichment Analysis of Differential Proteins. (d) After SOCS5 overexpression and knockdown, CD36 and FATP4 expressions were detected by Western blot in three HCC cell lines. The ratios indicate CD36 and FATP4 intensity, normalized to that of GAPDH. (e) The first 5 up-regulated and the first 5 down-regulated metabolites in positive mode. (f-g) volcano plot shows differential metabolites in negative mode and positive mode. Fold Change Analysis and Student's t test were used for variance analysis. (h-i) Metabolomics assays for targeted medium- and long-chain fatty acids in Huh7 cells (μg/10^7 cells).

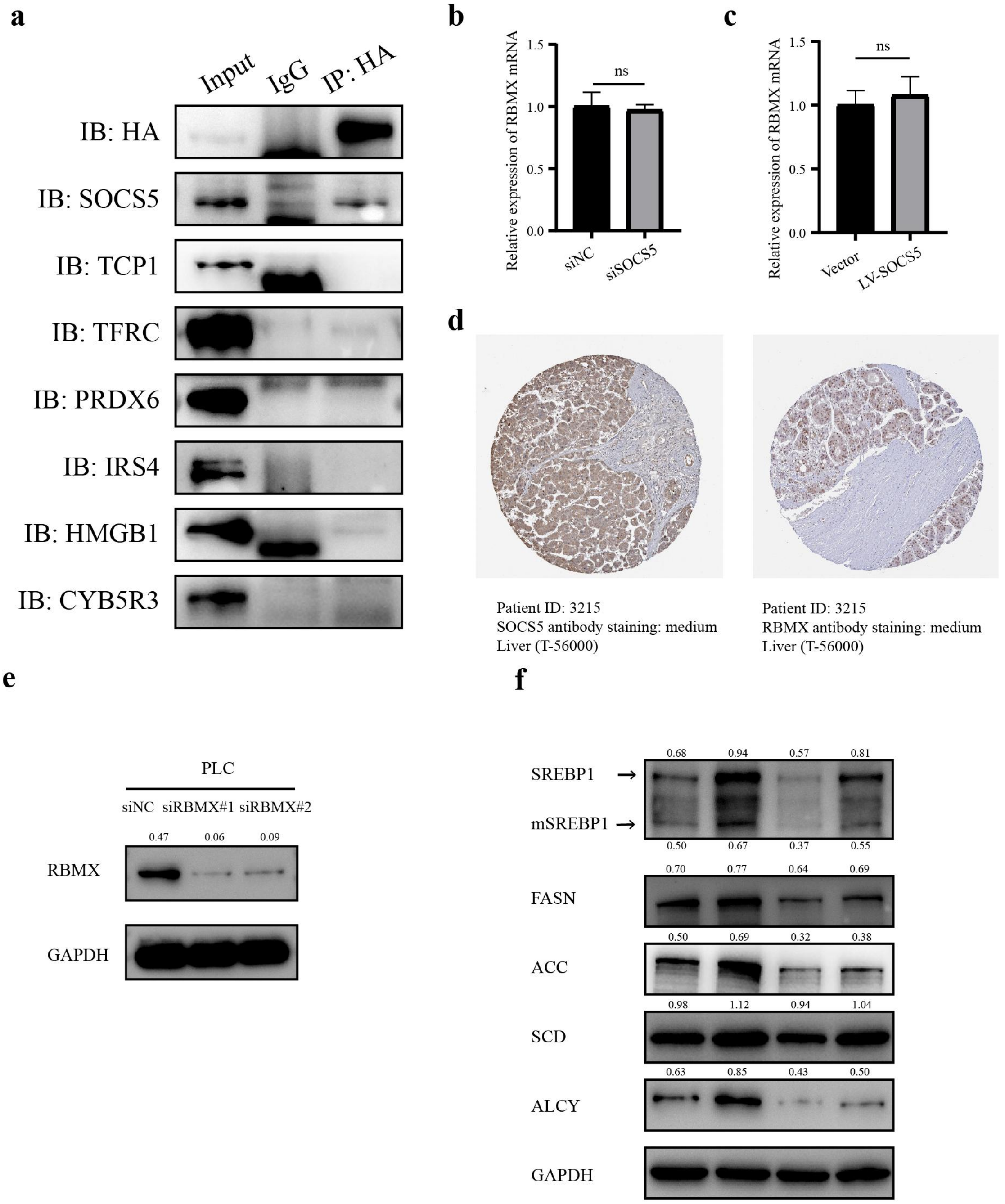

Supplementary Figure 7. Screen for interaction proteins of SOCS5. (a) Perform co-IP experiments to verify proteins that may interact with SOCS5. (b-c) SOCS5 has no significant regulation of RBMX at the mRNA level. P values were determined by Student's t test. (d) IHC results in Human Protein Atlas show that SOCS5 and RBMX are correlated at protein expression levels. (e) Western blot analysis of RBMX protein expression in Huh7 cells with siRBMX#1 and siRBMX#2 transfection. The ratios

indicate RBMX intensity, normalized to that of GAPDH. (f) Western blot analysis of SREBP1, mSREBP1, FASN, ACC, SCD and ACLY in Huh7 cells treated with LV-SOCS5 transfection and siRBMX#2 transfection.

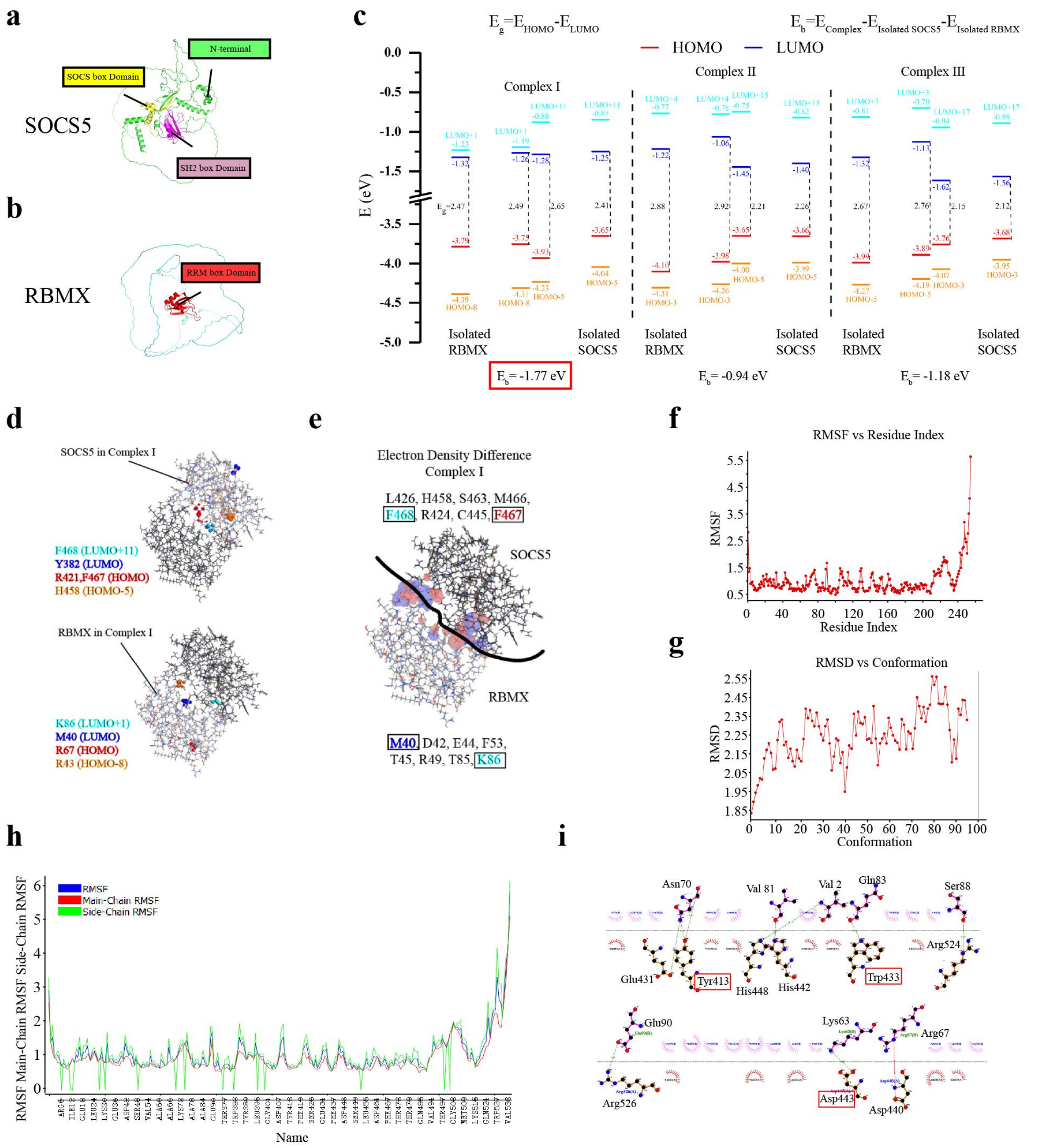

Supplementary Figure 8. Two prediction methods for SOCS5-RBMX complex models. (a-b) Schematic diagram of the alphafold structure of SOCS5 and RBMX. (c) Frontier molecular orbitals, HOMO and LUMO and important interface molecular

orbitals for three complex and isolated systems. (d) Frontier molecular orbitals, HOMO and LUMO and important interface molecular orbitals for complex I. (e) The electron density difference of complex I. (f) SOCS5-RBMX complex structure of Root Mean Square Deviation (RMSD). (g) Root Mean Square Fluctuation (RMSF) value of amino acid after simulation. (h) Main-Chain RMSF and Side-Chain RMSF in SOCS5-RBMX complex. (i) LigPlot draws the eyelash map of SOCS5-RBMX interaction.

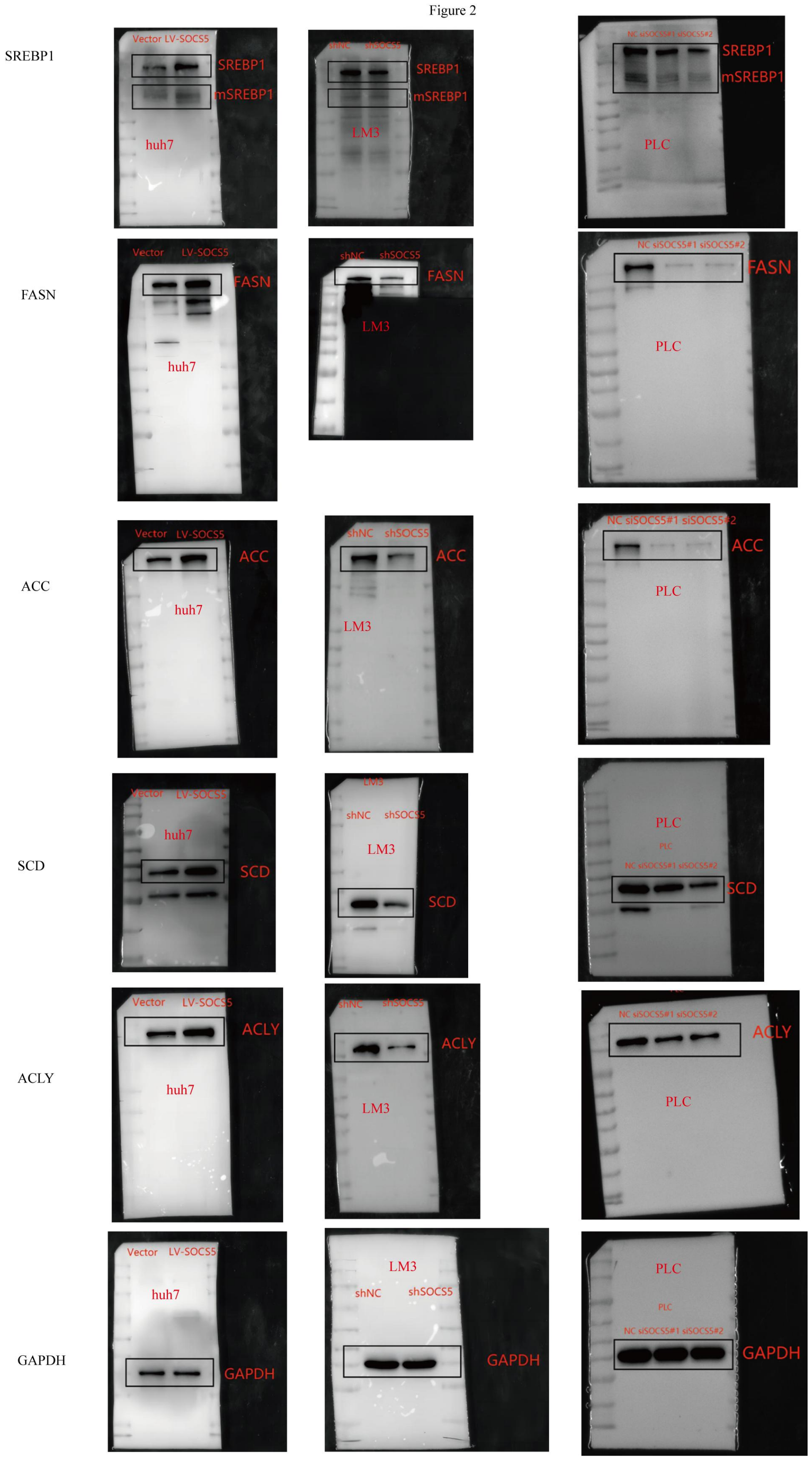

Supplementary Figure 9. Un-cropped scans of blots included in figure 2.

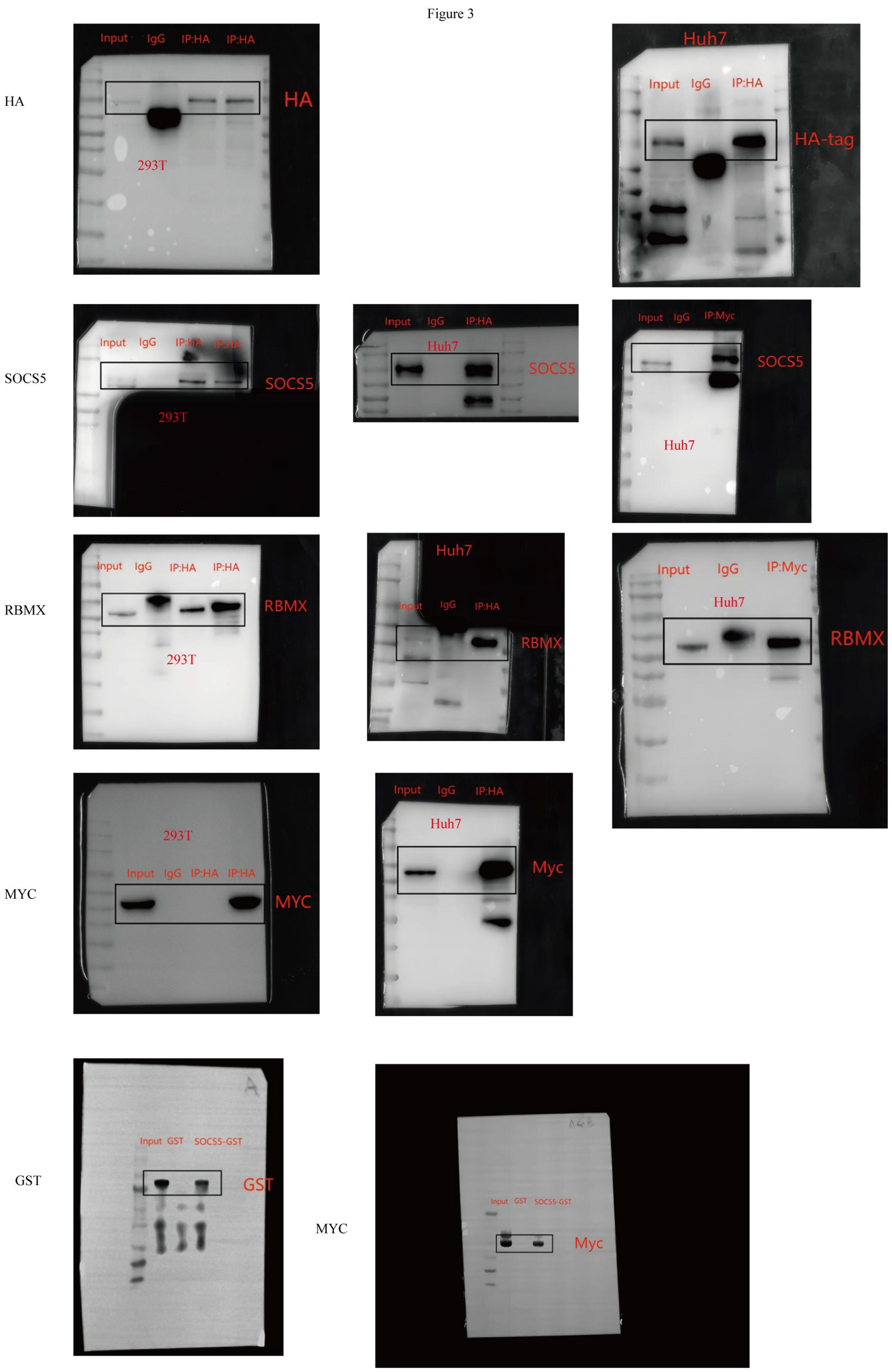

Supplementary Figure 10. Un-cropped scans of blots included in figure 3.

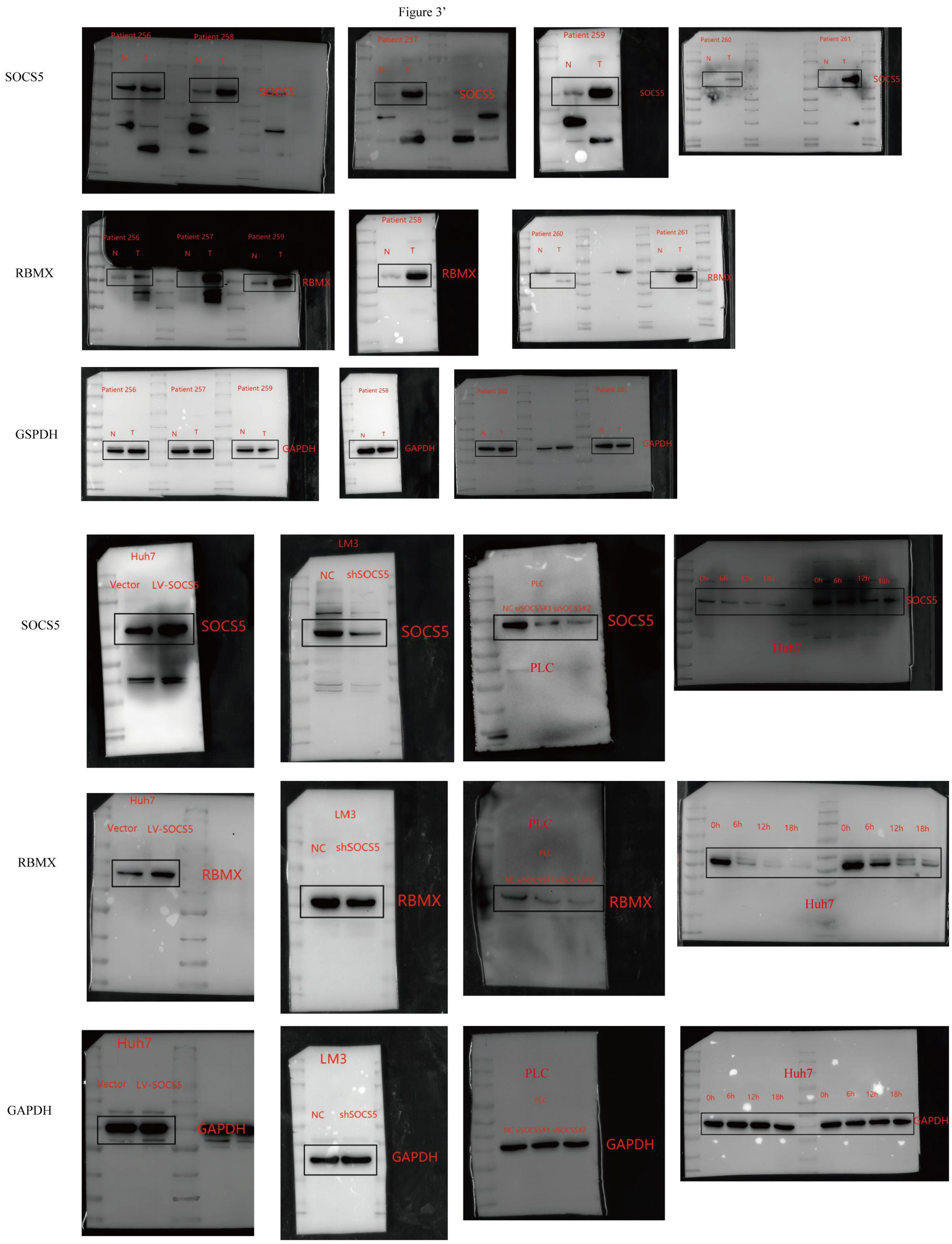

Supplementary Figure 11. Un-cropped scans of blots included in figure 3.

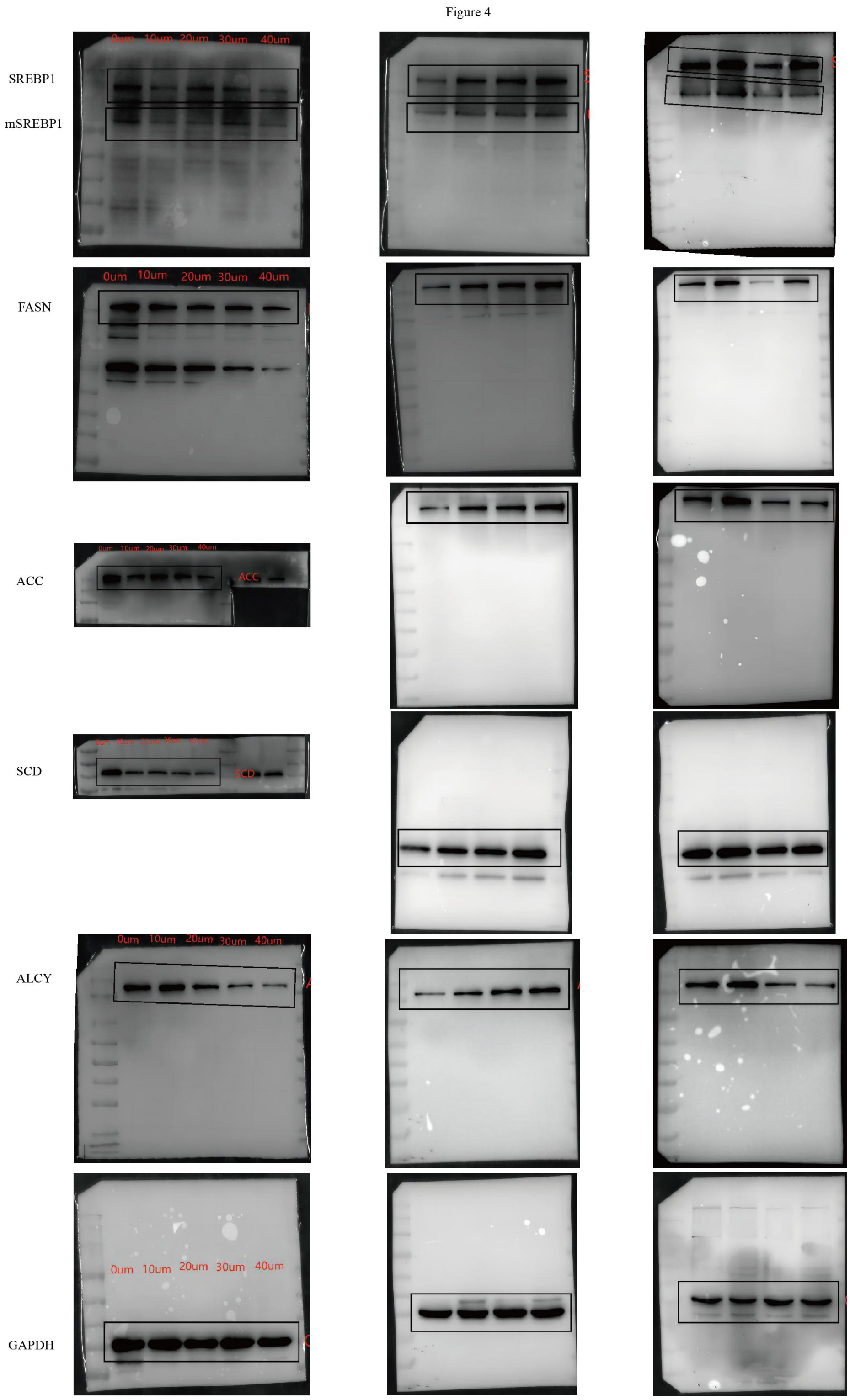

Supplementary Figure 12. Un-cropped scans of blots included in figure 4.

Figure 5

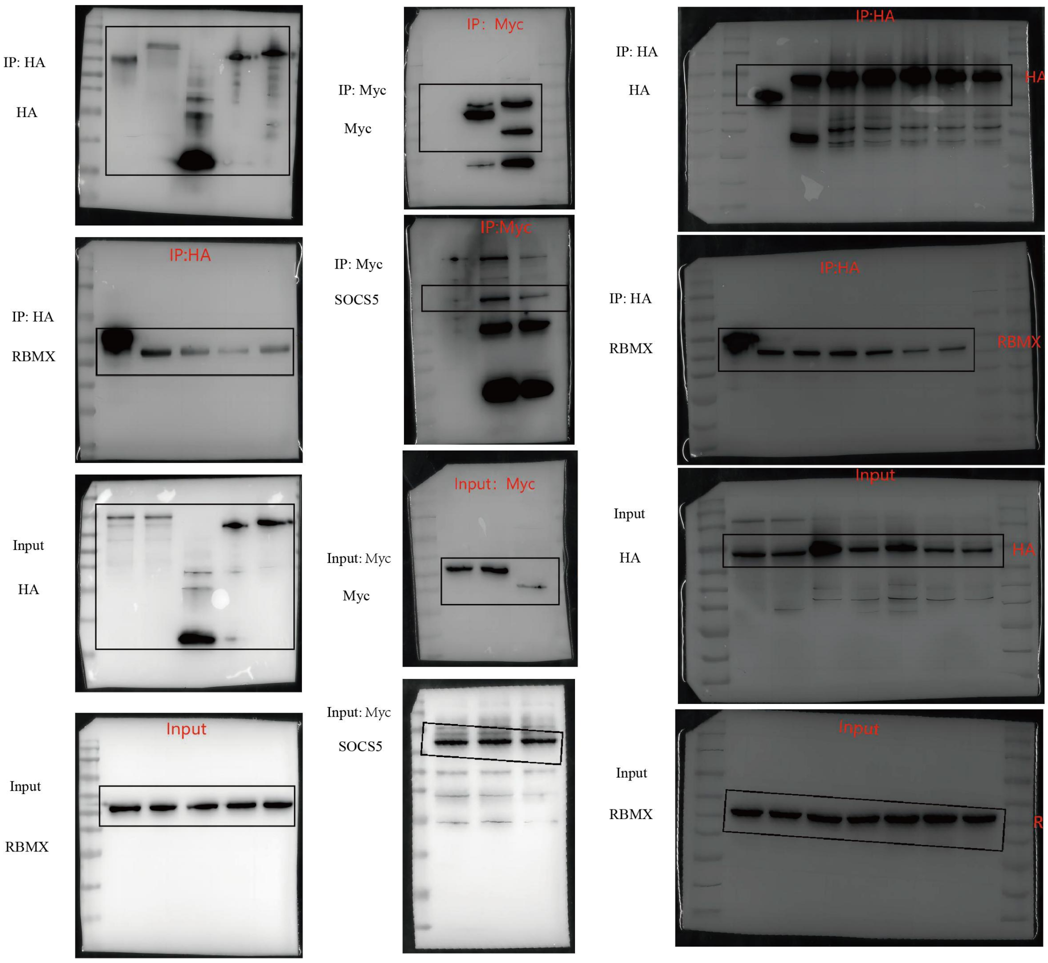

Figure 6

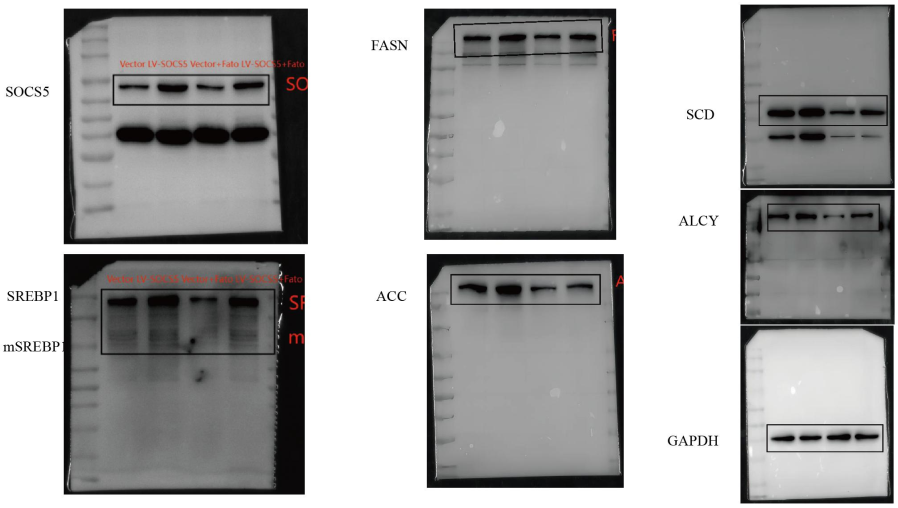

Supplementary Figure 13. Un-cropped scans of blots included in figure 5 and figure 6.

Supplementary Figure 6

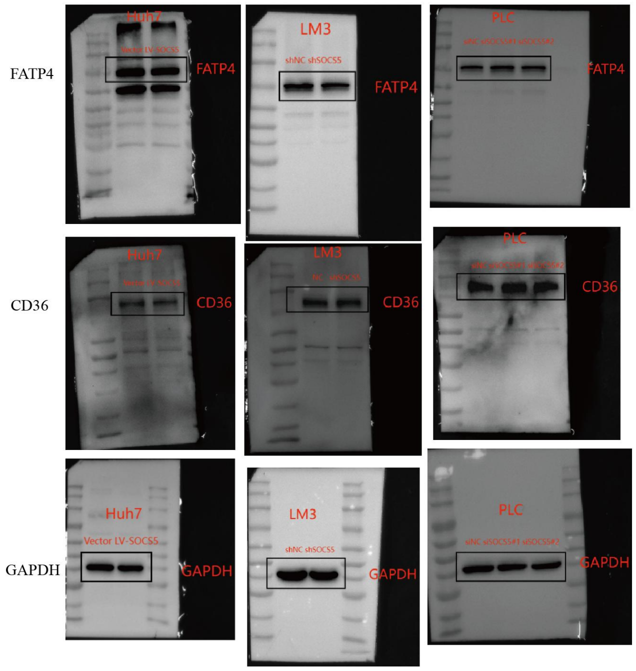

Supplementary Figure 7

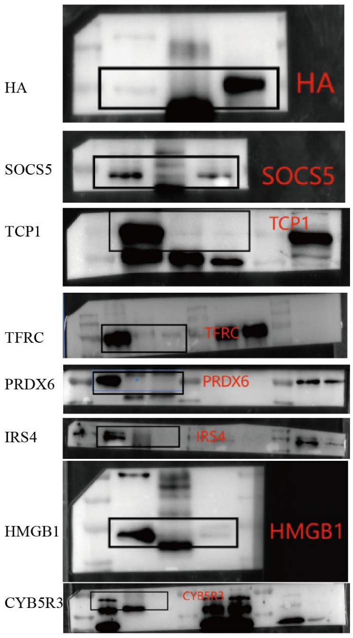

Supplementary Figure 7

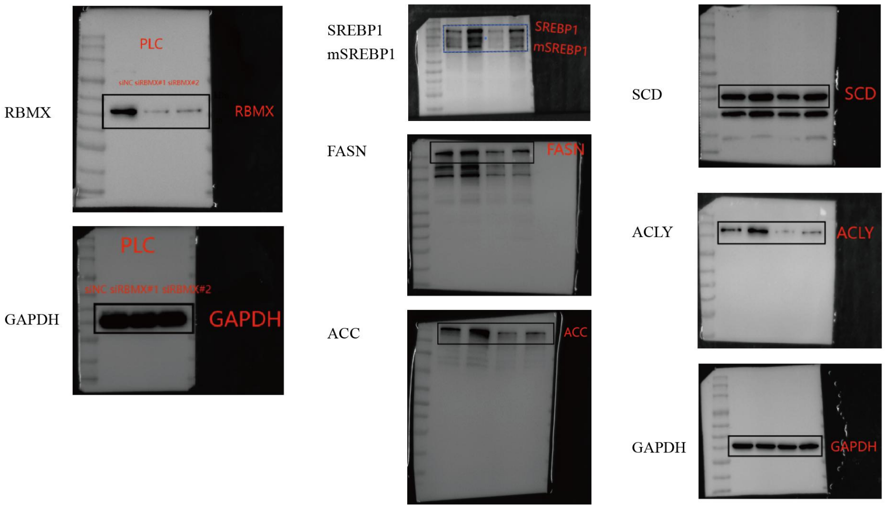

Supplementary Figure 14. Un-cropped scans of blots included in Supplementary Figure 6 and Supplementary Figure 7.

2. Supplement Tables

Supplementary Table 1. Correlation of steatosis with clinicopathological features between SBC-HCC and non SBC-HCC.

Items		Non SBC-HCC	SBC-HCC	χ^2	*P*
		175(81.0)	41(19.0)		
SOCS				6.881	**0.009****
	Low	91(52.0)	12(29.3)		
	High	84(48.0)	29(70.7)		
Differentiation				0.007	0.931
	I-II	114(65.1)	27(65.9)		
	III-IV	61(34.9)	14(34.1)		
Invasion of liver capsule				0.551	0.458
	No	70(40.0)	19(46.3)		
	Yes	105(60.0)	22(53.7)		
Tumor diameter				4.057	**0.044***
	≤ 3cm	77(44.0)	11(26.8)		
	> 3cm	98(56.0)	30(73.2)		
Gender				0.701	0.402
	Female	25(14.3)	8(19.5)		
	Male	150(85.7)	33(80.5)		
BMI				1.054	0.305
	≤ 24	70(40.0)	20(48.8)		
	> 24	105(60.0)	21(51.2)		
Tumor number				0.070	0.791
	1	142(81.1)	34(82.9)		
	⩾2	33(18.9)	7(17.1)		

Supplementary Table 2. Comparison of metabolic factors according to SBC-HCC or non SBC-HCC.

Serum index	SBC-HCC (n=41)	non SBC-HCC (n=175)	***P*** value
LDH	169.30±41.59	181.20±144.30	0.602
LDL-C	2.94±0.79	2.56±0.79	**0.004****
HDL-C	1.31±0.34	1.33±0.34	0.736
PA	234.10±66.18	215.6±60.33	0.085
TC	4.87±1.07	4.56±1.05	0.089
TG	1.11±0.50	0.92±0.43	**0.012***
TBA	18.45±20.66	11.62±12.68	**0.007****
TBIL	20.07±9.87	21.40±49.43	0.864
DBIL	6.74±3.06	8.90±36.68	0.706
IBIL	13.41±7.65	12.50±12.61	0.679
ALB	42.72±5.32	40.91±5.20	0.050
ALP	80.65±26.50	88.18±38.47	0.258
LPa	195.20±165.70	184.90±170.80	0.728

Supplementary Table 3. Comparison of metabolic factors according to SOCS5 protein expression level.

Serum index ($\bar{x}\pm S$)	Low SOCS5 (n=120)	High SOCS5 (n=125)	***P*** value
LDH	161.20±40.64	191.90±167.20	0.051
LDL-C	2.63±0.78	2.68±0.79	0.574
HDL-C	1.30±0.35	1.38±0.34	0.059
PA	224.40±58.40	224.1±69.80	0.964
TC	4.59±1.03	4.72±1.03	0.334
TG	0.99±0.54	0.97±0.53	0.771
TBA	10.66±8.86	14.38±17.37	**0.016** *
TBIL	17.13±7.47	23.80±58.16	0.258
DBIL	5.96±2.45	10.25±43.23	0.279
IBIL	23.82±132.10	26.51±146.20	0.880
ALB	41.16±4.79	41.47±5.36	0.642
ALP	83.71±29.26	93.70±63.23	0.123
LPa	180.50±190.60	205.30±179.70	0.300

Supplementary Table 4. Correlation of SOCS5 expression with clinicopathological features in HCC.

**Items**		**Low SOCS5**	**High SOCS5**	χ^2	***P***
		120(48.9)	125(51.1)		
Steatosis				9.921	**0.002****
	No	108(90)	94(74.6)		
	Yes	12(10)	31(25.4)		
Differentiation				2.534	0.111
	I-II	84(70)	76(60.3)		
	III-IV	36(30)	49(39.7)		
Invasion of liver capsule				1.207	0.272
	No	54(45)	48(38.1)		
	Yes	66(55)	77(61.9)		
Liver Cirrhosis				4.107	**0.043***
	No	48(40)	35(27.8)		
	Yes	72(60)	90(72.2)		
MVI				9.159	**0.002****
	No	97(80.3)	80(63.5)		
	Yes	23(19.1)	45(36.5)		
Tumor diameter				8.227	**0.004****
	≤ 5cm	96(80)	80(63.5)		
	> 5cm	24(20)	45(36.5)		
Gender				1.184	0.277
	Female	15(12.5)	22(17.5)		
	Male	105(87.5)	103(82.5)		
BMI				0.983	0.322
	≤ 24	44(36.7)	54(42.9)		
	> 24	76(63.3)	71(57.1)		
Fatty liver				1.014	0.314
	No	115(95.8)	116(92.9)		
	Yes	5(4.2)	9(7.1)		
HBV				0.581	0.446
	No	14(11.7)	11(8.7)		
	Yes	106(88.3)	114(91.3)		

3. Supplementary Methods

Public databases and Bioinformatics analysis

In the TCGA database (https://portal.gdc.cancer.gov/), RNA sequencing data (FPKM format) and diagnostic slides of 374 HCC patients were attained, as well as clinical data on patient age, survival time, tumor staging, etc. In addition, RNA-Seq gene expression profiles of Gao' HCC cohort (https://www.biosino.org/node/project/detail/OEP000321). RNA-Seq gene expression profiles of 107 HBV related HCC cohort from GSE121248 (https://www.ncbi.nlm.nih.gov). IHC images were obtained with the ProteinAtlas (https://www.proteinatlas.org/).

R 4.1.2 is used for subsequent analysis. R package "IOBR" to calculate the score of metabolism related signatures (https://github.com/IOBR/IOBR). Other R packages can be provided by the corresponding author.

Bioinformatics screening of driver genes for SBC-HCC

To identify the key genes involved in steatosis and lipid synthesis in HCC, we collected RNA-Seq gene expression profiles from 111 HBV-related cirrhosis HCCs in Gao' HCC cohort and 107 HBV related HCC cohort from GSE121248 (Supplementary Figure 4). We calculated the score of FA biosynthesis in Gao ' HBV-related cirrhosis HCC cohort and TCGA HCC cohort, and respectively divided the patients into Low lipid groups (n =55 in Gao; n = 53 in GSE121248) and High lipid groups (n = 56 in Gao; n = 54 in GSE121248). 3959 differentially expressed genes were identified in the Gao' HBV-related cirrhosis HCC cohort, and 1485

differentially expressed genes were identified in GSE121248. 261 common differential genes were identified ($p<0.05$; Supplementary Table 9). Subsequently, 261 common genes in the two HCC cohorts performed randomForest analysis, screening the top 50 important genes separately to obtain 9 common important genes (Supplementary Table 9). Univariate COX analysis of 9 common differential genes in Gao' HCC cohort identified 5 common genes with independent prognostic value ($p<0.05$). Further, 5 common prognosis-related genes were obtained by Kaplan-Meier analysis. Finally, 4 prognosis-related genes in the Gao' cohort performed randomForest analysis, and SOCS5 was identified as the most critical disease signature gene.

The PCR primers

SOCS5-F: ATTGATGGGCTCCCTCTACCC

SOCS5-R:TGCCTTGACTGGTTCTCGTTCC

GAPDH-F: TGACTTCAACAGCGACACCCA

GAPDH-R: CACCCTGTTGCTGTAGCCAAA

SCD-F: TTCGTTGCCACTTTCTTGCG

SCD-R: AAGTTGATGTGCCAGCGGTA

FASN-F: ACCTCCGTGCAGTTCTTGAG

FASN-R: GTTCAGGATGGTGGCGTACA

SREBP1-F: GTGCTTAGCCTCCTGACCTG

SREBP1-R: AGTTGTGTACCTTGTGGCCG

ACC-F: AGCCCTCAACAAAGTCCTCG

ACC-R: GGGCATGTAAGACAGCCAGT

RBMX-F: GGGCATGTAAGACAGCCAGT

RBMX-R: TGCCAAATACTGCTTCAAGAGC

ACLY-F: GATTTTGCGGGGTTCGTCG

ACLY-R: TCAGGAGTGACCCGAGCATA

Proteomics, Metabolomics and Liquid chromatography-mass spectrometry/mass spectrometry (LC-MS/MS)

Collect the same number ($1*10^7$) of stablely overexpressed SOCS5 or GV707 control HCC cells in 3 repeat cell samples. Samples are submitted to APTBIO (Shanghai, China) for Untargeted Metabolomics. Analyses were performed using an UHPLC (1290 Infinity LC, Agilent Technologies) coupled to a quadrupole time-of-flight (AB Sciex TripleTOF 6600) in Shanghai Applied Protein Technology Co., Ltd. The raw MS data (wiff.scan files) were converted to MzXML files using ProteoWizard MSConvert before importing into freely available XCMS software.

Similarly, Collect the same number ($1*10^7$) of three groups (overexpressed SOCS5 group, GV707 control group, overexpressed SOCS5 + Fatostain group) in 3 repeat cell samples. Samples are submitted to APTBIO (Shanghai, China) for targeting medium and long chain fatty acid metabolomics. The remaining steps are the same as above.

Collect the same number ($5*10^6$) of stablely overexpressed SOCS5 or GV707 control HCC cells. Samples are submitted to Shanghai Sinomics Corporation. HPLC Fractionation: the mix-sample was fractionated using a C18 column (Waters BEH

C18 4.6×250 mm, 5 μm) on a Rigol L3000 HPLC operating at 1 mL/min, the column oven was set as 50℃. LC-MS/MS Analysis-DDA mode: for transition library construction, shotgun proteomics analyses were performed using an U3000 UHPLC system (Thermo Fisher) coupled with an Orbitrap fusion mass spectrometer (Thermo Fisher) operating in the data-dependent acquisition (DDA) mode. Data analysis and visualization of DDA and DIA data were performed using Proteome Discoverer 2.4 (PD 2.4, thermo) platform, Biognosys Spectronaut version 13, and R statistical framework.

Huh7 HCC cells transfected with SOCS5-HA were immunoprecipitated with anti-HA antibodies or control IgG, and the pellet was submitted to Sangon Biotech (Shanghai, China) for LC-MS/MS. Mass spectrometry analysis was performed using the Q Exactive Plus LC/MS system (Thermo).

All cell samples are stored and transported in strict accordance with the company's requirements.
