# Supplementary material for: SOCS5-RBMX stimulates SREBP1-mediated lipogenesis to promote metastasis in steatotic HCC with HBV-related cirrhosis
Source: NPJ Precis Oncol. 2024 Mar 1;8:58. doi: 10.1038/s41698-024-00545-6 (PMC10907597; doi:10.1038/s41698-024-00545-6)
Supplement: Supplementary file 2 — Reporting Summary [file 41698_2024_545_MOESM2_ESM.pdf]

nature portfolio

Corresponding author(s): NPJPRECISIONONCOLOGY-03616R

Last updated by author(s): Jan 27, 2024

# Reporting Summary

Nature Portfolio wishes to improve the reproducibility of the work that we publish. This form provides structure for consistency and transparency in reporting. For further information on Nature Portfolio policies, see our Editorial Policies and the Editorial Policy Checklist.

## Statistics

For all statistical analyses, confirm that the following items are present in the figure legend, table legend, main text, or Methods section.

| n/a | Confirmed | |
|---|---|---|
| ☐ | ☒ | The exact sample size (*n*) for each experimental group/condition, given as a discrete number and unit of measurement |
| ☐ | ☒ | A statement on whether measurements were taken from distinct samples or whether the same sample was measured repeatedly |
| ☐ | ☒ | The statistical test(s) used AND whether they are one- or two-sided<br>*Only common tests should be described solely by name; describe more complex techniques in the Methods section.* |
| ☐ | ☒ | A description of all covariates tested |
| ☐ | ☒ | A description of any assumptions or corrections, such as tests of normality and adjustment for multiple comparisons |
| ☐ | ☒ | A full description of the statistical parameters including central tendency (e.g. means) or other basic estimates (e.g. regression coefficient) AND variation (e.g. standard deviation) or associated estimates of uncertainty (e.g. confidence intervals) |
| ☒ | ☐ | For null hypothesis testing, the test statistic (e.g. *F*, *t*, *r*) with confidence intervals, effect sizes, degrees of freedom and *P* value noted<br>*Give P values as exact values whenever suitable.* |
| ☒ | ☐ | For Bayesian analysis, information on the choice of priors and Markov chain Monte Carlo settings |
| ☒ | ☐ | For hierarchical and complex designs, identification of the appropriate level for tests and full reporting of outcomes |
| ☐ | ☒ | Estimates of effect sizes (e.g. Cohen's *d*, Pearson's *r*), indicating how they were calculated |

*Our web collection on statistics for biologists contains articles on many of the points above.*

## Software and code

Policy information about availability of computer code

| | |
|---|---|
| Data collection | TCGA HCC RNA-seq data and diagnostic slides of 374 HCC patients were downloaded from TCGA dataset (https://portal.gdc.cancer.gov/), as well as clinical data on patient age, survival time, tumor staging, etc. In addition, RNA-Seq gene expression profiles of Gao' HCC cohort (https://www.biosino.org/node/project/detail/OEP000321). RNA-Seq gene expression profiles of HBV-related HCC cohort from GSE121248 (https://www.ncbi.nlm.nih.gov). IHC images were obtained with the ProteinAtlas (https://www.proteinatlas.org/). |
| Data analysis | SPSS 16.0 and GraphPad 8.0 were used for statistical analysis. R 4.2.1 is used for subsequent analysis. R package "IOBR" to calculate the score of metabolism related signatures (https://github.com/IOBR/IOBR). Custom code or scripts in the generation or analysis of datasets are available from the corresponding author upon request. |

For manuscripts utilizing custom algorithms or software that are central to the research but not yet described in published literature, software must be made available to editors and reviewers. We strongly encourage code deposition in a community repository (e.g. GitHub). See the Nature Portfolio guidelines for submitting code & software for further information.

## Data

Policy information about availability of data

All manuscripts must include a data availability statement. This statement should provide the following information, where applicable:

- Accession codes, unique identifiers, or web links for publicly available datasets
- A description of any restrictions on data availability
- For clinical datasets or third party data, please ensure that the statement adheres to our policy

TCGA HCC RNA-seq data and diagnostic slides of 374 HCC patients were downloaded from TCGA dataset (https://portal.gdc.cancer.gov/), as well as clinical data on patient age, survival time, tumor staging, etc. In addition, RNA-Seq gene expression profiles of Gao' HCC cohort (https://www.biosino.org/node/project/detail/OEP000321). RNA-Seq gene expression profiles of HBV-related HCC cohort from GSE121248 (https://www.ncbi.nlm.nih.gov). IHC images were obtained with the ProteinAtlas (https://www.proteinatlas.org/).
The data of proteomics data generated in this study can be viewed in the Integrated Proteome Resources (https://www.iprox.cn/) database (accession no. PXD048740). All relevant data are available from the authors upon request.

## Research involving human participants, their data, or biological material

Policy information about studies with human participants or human data. See also policy information about sex, gender (identity/presentation), and sexual orientation and race, ethnicity and racism.

| | |
|---|---|
| Reporting on sex and gender | A total of 261 HCC samples were collected from the Affiliated Hospital of Qingdao University, including 225 males and 36 females. The sex and gender of these patients are consistent. We provide details of these patients in Table S5. |
| Reporting on race, ethnicity, or other socially relevant groupings | These human participants are all from China. Over 95% of human participants were Han Chinese, which is a predominant ethnic population in China. These race and ethnicity characteristics are defined by the Chinese Resident Identification Information issued by the Chinese government |
| Population characteristics | We provide details of these patients in Table S5. |
| Recruitment | From January 2013 to December 2016, 245 consecutive HCC patients from the Affiliated Hospital of Qingdao University were recruited. An additional 16 frozen HCC tissues and matched adjacent non-tumor liver tissues were collected from patients who underwent surgery at the Affiliated Hospital of Qingdao University in 2022. |
| Ethics oversight | This study was approved by the Institutional Review Board of the Affiliated Hospital of Qingdao University (QYFYWZLL27315). All patients provided written informed consent to participate. |

Note that full information on the approval of the study protocol must also be provided in the manuscript.

# Field-specific reporting

Please select the one below that is the best fit for your research. If you are not sure, read the appropriate sections before making your selection.

☒ Life sciences ☐ Behavioural & social sciences ☐ Ecological, evolutionary & environmental sciences

For a reference copy of the document with all sections, see nature.com/documents/nr-reporting-summary-flat.pdf

# Life sciences study design

All studies must disclose on these points even when the disclosure is negative.

| | |
|---|---|
| Sample size | The inclusion criteria were as follows: age ≥18 years; Child–Pugh grade A; primary surgical resection of HCC; pathologically confirmed HCC, including single or multiple tumors; and no hilar lymph node involvement or extrahepatic metastasis. |
| Data exclusions | The inclusion criteria were as follows: age ≥18 years; Child–Pugh grade A; primary surgical resection of HCC; pathologically confirmed HCC, including single or multiple tumors; and no hilar lymph node involvement or extrahepatic metastasis. |
| Replication | All attempts at replication were successful. |
| Randomization | Consecutive HCC patients from the Affiliated Hospital of Qingdao University were recruited. |
| Blinding | The investigators were blinded to group allocation during data collection. Consecutive HCC patients who met the inclusion criteria from the Affiliated Hospital of Qingdao University were recruited. |

# Reporting for specific materials, systems and methods

We require information from authors about some types of materials, experimental systems and methods used in many studies. Here, indicate whether each material, system or method listed is relevant to your study. If you are not sure if a list item applies to your research, read the appropriate section before selecting a response.

## Materials & experimental systems

| n/a | Involved in the study |
|---|---|
| ☐ | ☒ Antibodies |
| ☒ | ☐ Eukaryotic cell lines |
| ☒ | ☐ Palaeontology and archaeology |
| ☐ | ☒ Animals and other organisms |
| ☐ | ☒ Clinical data |
| ☒ | ☐ Dual use research of concern |
| ☒ | ☐ Plants |

## Methods

| n/a | Involved in the study |
|---|---|
| ☒ | ☐ ChIP-seq |
| ☒ | ☐ Flow cytometry |
| ☒ | ☐ MRI-based neuroimaging |

## Antibodies

| | |
|---|---|
| Antibodies used | SOCS5 (sc-100858, Santa Cruz); HA-Tag (#3724, CST); hnRNP G (ab190352, Abcam); FASN (#3180, CST); Myc-Tag (#2276, CST); ACC (#3676, CST); SCD1 (ab236868, Abcam); ACLY (ab40793, Abcam); SREBP1 (14088-1-AP, proteintech); IgG (#2729, CST); HRP anti-mouse IgG (abs20001, absin); HRP anti-rabbit IgG (abs20002, absin); CD36 (ab252922, abcam); FATP4(ab200353, abcam). |
| Validation | Validation of each primary antibody for the species and application according to the antibody-related information on the manufacturer's website. |

## Animals and other research organisms

Policy information about studies involving animals; ARRIVE guidelines recommended for reporting animal research, and Sex and Gender in Research

| | |
|---|---|
| Laboratory animals | 20 male C57BL/6J mice (4 weeks old) and 52 male BALB/nude mice (4 weeks old) were included in this study. |
| Wild animals | All animals were purchased from Beijing Vital River Laboratory Animal Technology (Beijing, China), and transported by car to the Animal Center of the Affiliated Hospital of Qingdao University. After the experiments, the anesthetized mice were sacrificed by cervical dislocation. |
| Reporting on sex | The mice used in this study were all males. |
| Field-collected samples | All animals were housed, maintained, and treated (as described in Supplementary Materials and Methods) in a SPF barrier environment that was maintained at a constant temperature (23–25 °C) and humidity (50–60%), and under 12 h light / dark cycle. The end-of-experiment protocol is described in detail in the Supplementary Materials and Methodology. |
| Ethics oversight | The treatment of mice was carried out in strict accordance with the principles approved by the Animal Experimental Ethics Committee of the Affiliated Hospital of Qingdao University. |

Note that full information on the approval of the study protocol must also be provided in the manuscript.

## Clinical data

Policy information about clinical studies

All manuscripts should comply with the ICMJE guidelines for publication of clinical research and a completed CONSORT checklist must be included with all submissions.

| | |
|---|---|
| Clinical trial registration | none |
| Study protocol | none |
| Data collection | none |
| Outcomes | none |

## Plants

| | |
|---|---|
| Seed stocks | none |
| Novel plant genotypes | none |
| Authentication | none |
